# Design and synthesis of novel pyrimidine-pyrazole hybrids with dual anticancer and anti-inflammatory effects targeting BRAFV600E and JNK

**DOI:** 10.1007/s11030-025-11121-w

**Published:** 2025-02-22

**Authors:** Mohammed S. Abdel-Maksoud, Hebatollah E. Eitah, Rasha M. Hassan, Walaa Hamada Abd-Allah

**Affiliations:** 1 Medicinal and Pharmaceutical Chemistry Department, Pharmaceutical and Drug Industries Research Institute, National Research Centre (NRC), (ID: 60014618), Dokki, P.O. 12622, Giza, Egypt; 2 Medicinal and Pharmaceutical Chemistry Department, Pharmaceutical and Drug Industries Research Institute, National Research Centre (NRC) (Pharmacology Group), (ID: 60014618), Dokki, P.O. 12622, Giza, Egypt; 3 Pharmaceutical Chemistry Department, Collage of Pharmaceutical Science and Drug Manufacturing, Misr University for Science and Technology, P.O. 77, 6th of October City, Giza, Egypt

**Keywords:** Antiproliferative, Anti-inflammatory, JNK kinase inhibitors, Pyrazole, Sulfonamide

## Abstract

Two new series of pyrimidinyl ethyl pyrazoles derivatives **13a–f** and **14a–f** were designed and synthesized to possess both anticancer effect by inhibiting BRAFV600E and anti-inflammatory effect by inhibiting JNK isoforms. The structure of the new compounds was generated from hybridization of two main moieties. The pyrimidinyl moiety from reported BRAFV600E inhibitors, and the pyrazole moiety from JNK isoforms inhibitors. The new final compounds were tested on BRAFV600E, JNK1, JNK2, and JNK3 to measure their kinases inhibitory effect. Compound **14c** showed the highest activity on JNK isoforms and BRAFV600E with IC_50_ = 0.51 μM, 0.53 μM, 1.02 μM, 0.009 μM on JNK1, JNK2, JNK3,and BRAFV600E, respectively. All final compounds were tested over four cancer cell lines related to the target enzymes. Compound **14d** showed the most potent activity on all tested cell lines with IC_50_ = 0.87 μM, 0.91, 0.42 μM and 0.63 μM on MOLT-4, K-562, SK-MEL-28, and A375 cell lines, respectively. The ability of **14d** and **14c** to inhibit MEK1/2 and ERK1/2 phosphorylation was performed by using western blot. The cell cycle analysis of compound **14d** on A375 cell line revealed that compound **14d** arrested cell growth at G0-G1 phase. Compound **14d** remarkably decreased cell migration compared to control group in traditional migration test. Compounds **13a–f** and **14a–f** showed significant ability to inhibit nitric oxide release and PGE2 production on raw 264.7 macrophages. Compounds **13d** and 1**4d** exhibited high inhibitory effect on iNOS and COX-2 compared to COX-1. Finally, the effect of most potent compounds on TNF-alpha and IL-6 was determined.

**Graphical abstract:**

Two new series of pyrimidinyl ethyl pyrazoles were synthesized. The new derivatives were able to inhibit both JNK isoforms and BRAFV600E. JNK and BRAFV600E play a key role in both cancer and inflammatory disorders. Final compounds were tested for their anticancer and anti-inflammatory activities.

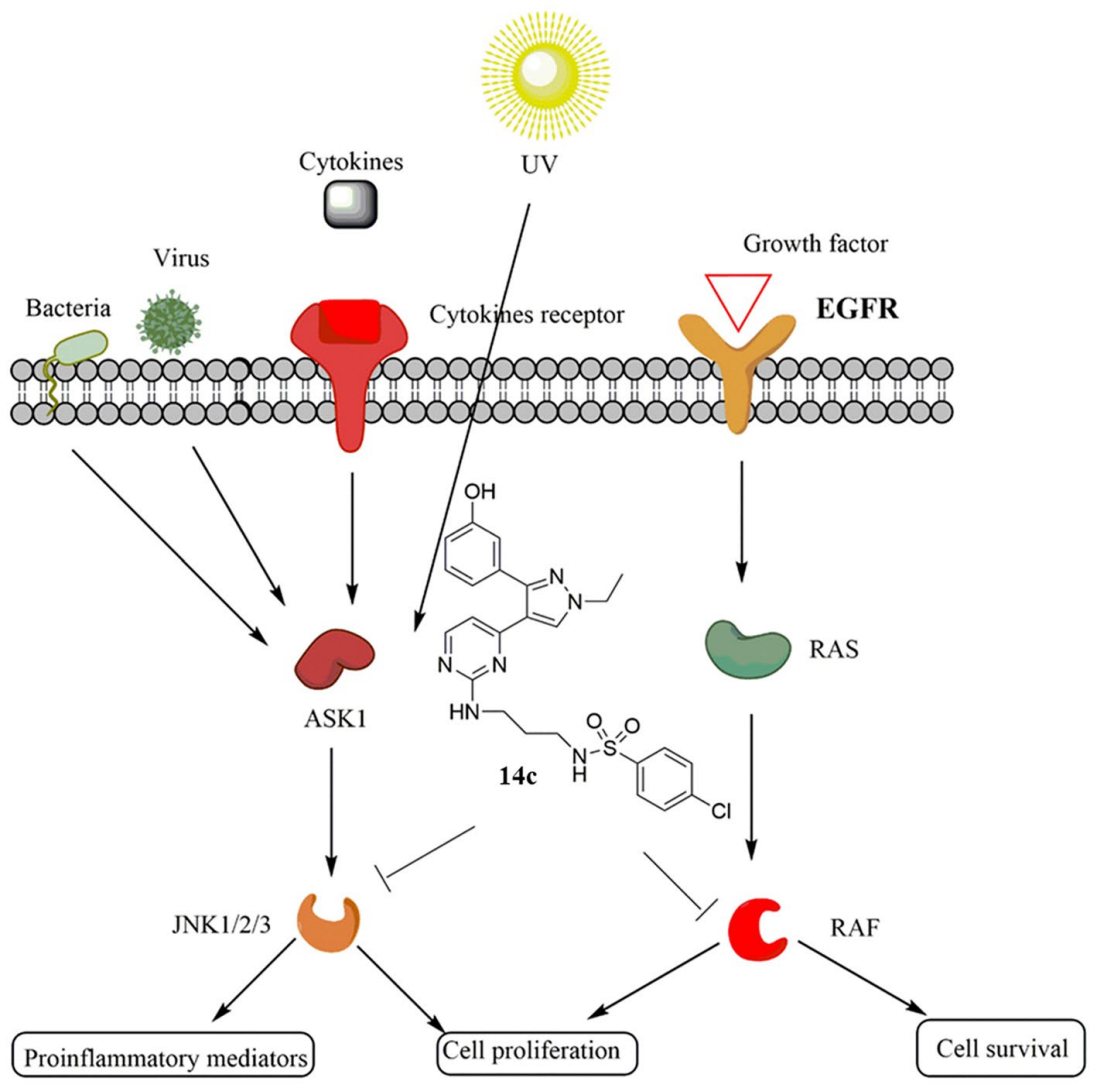

**Supplementary Information:**

The online version contains supplementary material available at 10.1007/s11030-025-11121-w.

## Introduction

Cancer and inflammatory disorders are considered as the oldest known causes of health problems and deaths worldwide [1–3]. Regarding cancer, recent data expected that in 2024 about 2 million new cases and 600.000 death cases will be exist in the United States only [4]. The world health organization announced that in 2022 there were 20 million new cancer cases and 9.7 million cancer related deaths. On the other hand, inflammation which is the main defense mechanism in human body could be fatal in many situations. Inflammatory disorders affect 20–30% of human population [5]. In 2020 Covid-19 virus caused 7 million human death cases which were related to inflammatory reactions due to viral infection that led to cytokines storm which is considered the main reason for viral mortality [6].

The first connection between cancer and inflammation was reported by Rudolf Virchow in nineteenth century who found leukocytes at cancer tissues [7]. The role of inflammation on tumorigenesis was confirmed in many studies [7]. In case of chronic inflammation, the environment around and within inflammatory area which has high content of oxygen, nitrogen, cytokines, and different types of immune cells could exacerbate damage of DNA and consequent atypical cell production that leads to cancer [8–10].

Intracellular signal transduction, which leads to cell division, differentiation, migration, and death, is the process by which signals from the cell surface are sent to the nucleus in essential cellular processes. Protein kinases phosphorylate about 30% of the proteins found in cells.The transfer of a gamma phosphate group from ATP to an acceptor amino acid (tyrosine, serine, or threonine) in a substrate protein is catalyzed by protein kinases [11, 12]. Several disorders such as cancer and inflammatory disorders are brought on by abnormal regulation, aberrant activation, genetic changes and/or mutations in protein kinases. These factors can also result in resistance to anti-cancer medications [13–15].

The traditional therapies of both cancer and inflammation cause many side effects. Traditional cancer chemotherapies are non-selective and all patients are suffering from anxiety, taste changes, mouth sores, vomiting, nausea, increased satiety, pain, depressed mood, and fatigue [16]. Similarly, the main treatments of inflammation include corticosteroids and non-steroidal anti-inflammatory drugs and both types provoke many physiological disorders which may threaten patient’s life such as elevated risk of infection, psychiatric disorders, liver damage, kidney failure, and gastrointestinal ulcers [17–19].

At the end of the twentieth century, two main millstones paved the way for new era for both cancer and inflammatory treatment. The first millstone in 1980 was the discovery of protein kinase inhibition which increased the ability to produce more precise and high selective therapies for kinases dysfunction related diseases. The second milestone was the discovery of eukaryotic whole human genome that contains 518 protein kinases which allow scientists to find a plethora of biological targets for each disease [20]. MAPK are a group of serine/threonine kinases which control cell growth and functions [21]. BRAF and JNK are main components of MAPK family and any change of their level or activity rate was associated with many diseases. BRAF hyperactivation occurs as a result of enzyme mutation. BRAFV600E is the most common mutation in BRAF where valine is replaced by glutamate at residue 600. This mutated form of the enzyme is responsible for many cancer types such as melanoma, thyroid gland cancer, and colon cancer [22]. In the United States and the European Union, vemurafenib was the first approved drug in 2011 and 2012, respectively for the treatment of BRAF-mutated metastatic melanoma [23]. On the Other hand, JNK isoforms are activated by inflammatory mediators such as cytokines and stress conditions. Hyperactivation of JNK was related to many human diseases such as inflammation and cancer [24].

Targeting multiple kinases is a more effective approach than targeting a single kinase because cancer is regulated by multiple pathways that can compensate for one another when a single route is blocked. Furthermore, there are many advantages associated with multi-kinase inhibition, including increased efficacy as a result of its synergistic effect, decreased likelihood of polypharmacy toxicity, avoidance of the pharmacokinetic incompatibilities and improved selectivity [25, 26].

**Fig. 1:**
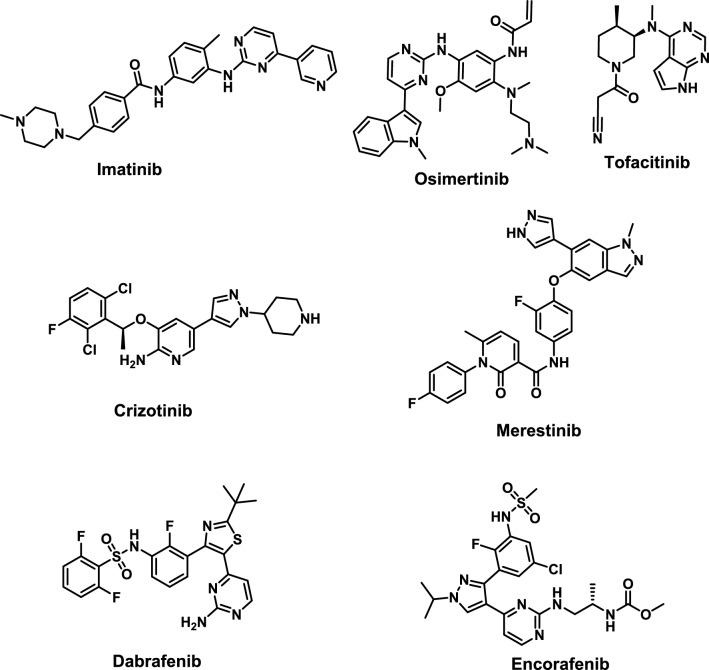
Structures of some protein kinase inhibitors having pyrimidine and pyrazole moieties

Some pyrimidine derivatives that have protein kinase inhibitory effects have been clinically approved as chemotherapeutic agents such as imatinib and osimertinib (Fig. 1) which are used for the treatment of leukemia and non-small lung cancer, respectively [27]. Also, tofacitinib is a pyrimidine based anti-inflammatory drug that acts by inhibition of protein kinase and approved for the treatment of rheumatoid arthritis, psoriatic arthritis and ulcerative colitis [28]. In addition, pyrazole and its derivatives have displayed broad biological activities as anticancer [29] and anti-inflammatory effects [30]. Moreover, several pyrazole-containing multi-kinase inhibitors have been approved for clinical use for treatment of cancer such as crizotinib or currently in clinical trials as merestinib [31].

On the other hand, sulfonamide is a preferred scaffold that has been adding several bioactive candidates with a wide range of varied biological activities to the medicinal chemistry library [32–34]. Many of anticancer sulfonamides that have been described work by inhibiting protein kinases; some of these are FDA-approved or undergoing clinical studies. Dabrafenib is a pyrimidine benzenesulfonamide hybrid drug which was approved for treatment of cancers linked to mutated BRAFV600E [35]. Also, the pyrazole-pyrimidine sulfonamide hybrid encorafenib which has BRAFV600E inhibitory efficacy, received approval for the treatment of resistant melanoma when used in conjunction with binimetinib [36].

**Fig. 2:**
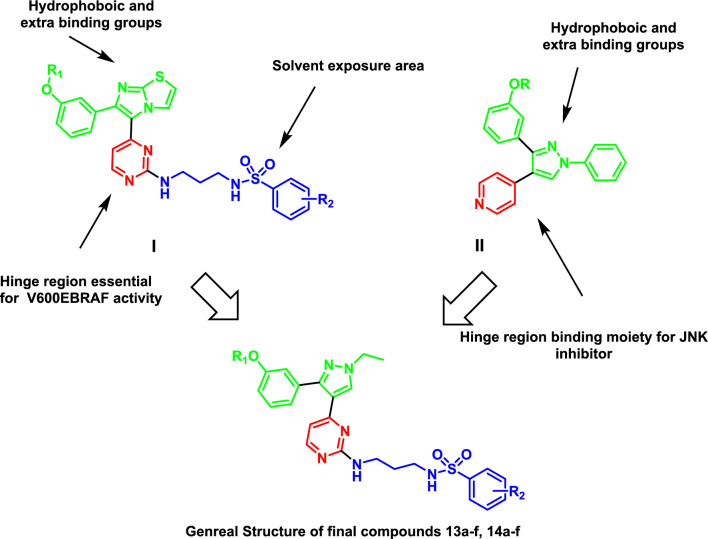
Rational design of present work

Currently, one of the approaches used to find new scaffolds of dual or multi-targeted small molecule inhibitors is the molecular hybridization approach [37–40]. In our previous work a plethora of pyrimidinyl sulfonamide derivatives were synthesized and investigated for their BRAFV600E activity [41–46]. Compounds with general structure **I** (Fig. 2) showed high inhibitory activity on BRAFV600E but did not exhibit high activity on JNK isoforms. On the other hand, pyrazole containing compounds with general structure **II** showed moderate anticancer and anti-inflammatory effects with moderate activity on JNK isoforms and BRAFV600E [47]. In the present work, fragment based drug design was applied to design a new hybrid between the pyrimidine sulfonamide terminal moiety from compound **I** and 3-phenyl pyrazole scaffold from compound **II**. The designed compounds are expected to fit in the three primary sections make up the binding site of kinase inhibitors: the hydrophobic rear cleft, the gate area, and the hinge region (front cleft). The target compounds were successfully synthesized. Both anticancer and anti-inflammatory effects of final derivatives were investigated.

## Results and discussion

### Chemistry

**Scheme 1:**
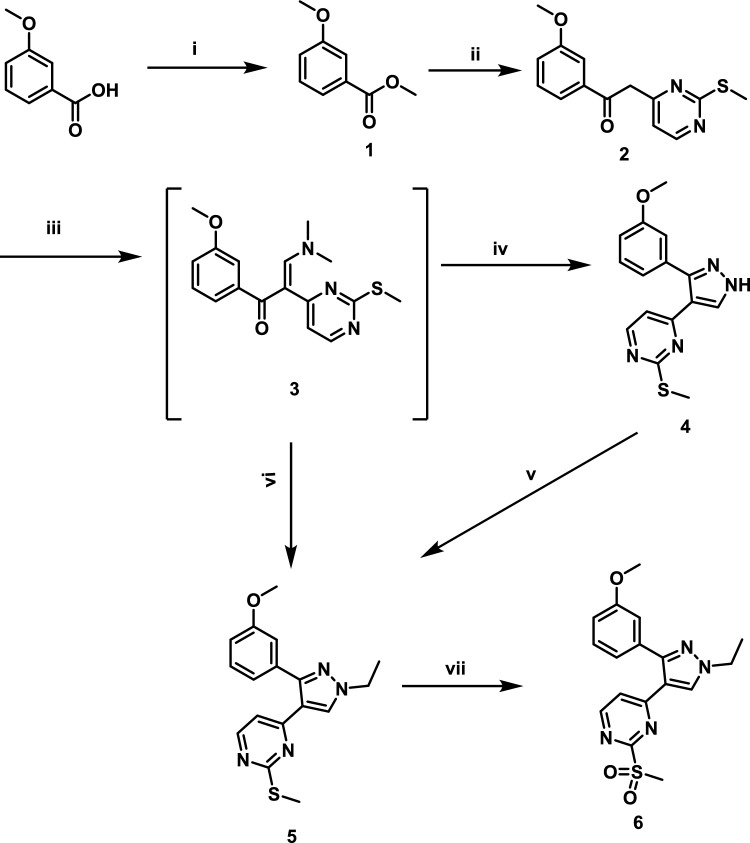
Synthetic pathway of main intermediate **6,** Reagents and conditions: (i) Conc. sulfuric acid, methanol, reflux; (ii) 4-Methyl-2-(methylthio)pyrimidine, LHMDS, THF, 0 °C, overnight; (iii) DMF-DMA, reflux, overnight; (iv) Hydrazine, methanol, rt; (v) Ethyl iodide, DMF, NaH, rt, (vi) Ethyl hydrazine, methanol, rt, 16 h, (vii) Oxone, methanol, H_2_O, 16 h

The synthetic process of final target compounds started from the synthesis of 4-(1-ethyl-3-(3-methoxyphenyl)-1H-pyrazol-4-yl)-2-(methylsulfonyl)pyrimidine (**6**) as key intermediate to produce final compounds. Starting from meta anisic acid which was subjected to esterification by refluxing in methanol and using concentrated sulfuric acid as catalyst. The formed methyl 3-methoxybnzoate (**1**) was coupled with 4-methyl-2-(methylthio)pyrimidine using lithium 1,1,1-trimethyl-*N*-(trimethylsilyl)silanaminide as a strong base to form 1-(3-methoxyphenyl)-2-(2-(methylthio)pyrimidin-4-yl)ethan-1-one (**2**). Compound **2** was refluxed in 1,1-dimethoxytrimethylamine to give 3-(dimethylamino)-1-(3-methoxyphenyl)-2-(2-(methylthio)pyrimidin-4-yl) prop-2-en-1-one (**3**). Pyrazole ring was formed by reaction of compound **3** with hydrazine hydrate in methanol to give 4-(3-(3-methoxyphenyl)-1*H*-pyrazol-4-yl)-2-(methylthio)pyrimidine (**4**) which was converted to 4-(1-ethyl-3-(3-methoxyphenyl)-1*H*-pyrazol-4-yl)-2-(methylthio)pyrimidine (**5**) via reaction with ethyl iodide in DMF and using sodium hydride. Compound **5** could be produced directly from compound **3** by reacting with ethyl hydrazine in methanol (ethyl hydrazine hydrochloride in methanol and potassium carbonate) to produce 4-(1-ethyl-3-(3-methoxyphenyl)-1*H*-pyrazol-4-yl)-2-(methylthio)pyrimidine (**5**). Compound **5** was converted to the desired intermediate **6** by using potassium peroxysulfate Scheme 1.

**Scheme 2:**
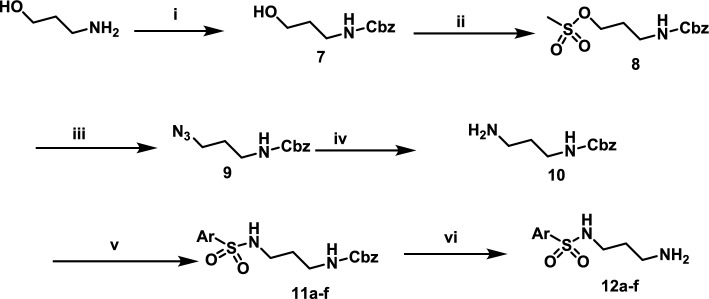
Synthetic pathway of side chains **12a–f**, Reagents and conditions: (i) Benzylchloroformate, triethylamine, dichloromethane, 0 °C, (ii) Methane sulfonyl chloride, triethylamine, 3h, rt, (iii) NaN_3_, DMSO, 80 °C, 3h.; (iv) Triphenylphosphine, methanol, reflux, 12 h; (v) Appropriate sulfonyl chloride, TEA, dichloromethane, rt, overnight; (vi) Pd/C, H_2_, methanol, rt, 3–6 h

In the second part of the planned synthetic route, formation of linker and terminal sulfonamide derivatives were performed. Starting from 3-aminopropan-1-ol that was protected by Cbz to give benzyl (3-hydroxypropyl)carbamate (**7**). Compound **7** was converted to 3-(((benzyloxy)carbonyl)amino)propyl methanesulfonate (**8**) by reaction with methansulfonyl chloride in dichloromethane and using triethylamine as a base at 0 °C. The formed mesylate ester was converted to benzyl (3-azidopropyl)carbamate (**9**) by heating compound **8** with sodium azide in DMSO and heat to 80 °C for 3 h. Reduction of compound **9** using triphenyl phosphine in methanol converted it to protected diamino compound **10**. Compound **10** was reacted with appropriate sulfonyl chloride in dichloromethane and in presence of diisopropylethyl amine to give N protected side chains **11a–f**. Removal of protection group by Pd/carbon under hydrogen atmosphere produced the wanted side chains **12a–f** Scheme2.

**Scheme 3:**
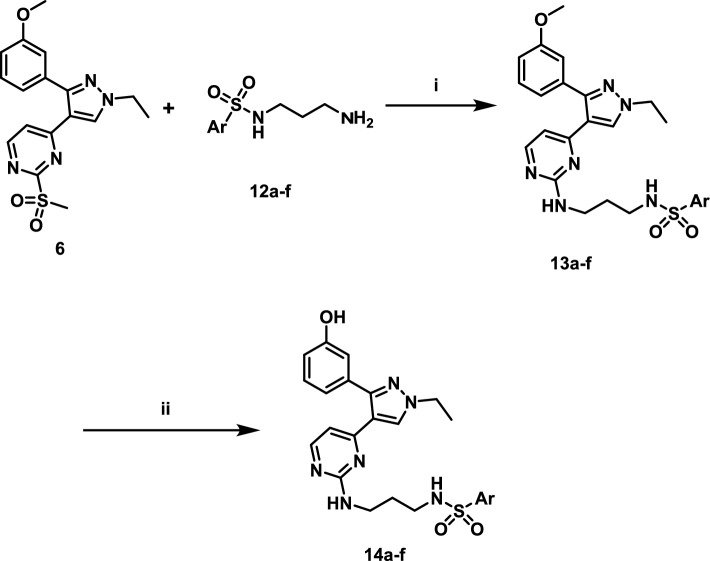
Synthesis of final target compounds. Reagents and conditions: (i) DPIA, DMSO, 80 °C, 8h; (ii) BBr_3_, dichloromethane, 0 °C

The final steps to obtain final target compounds were presented in scheme 3. The main intermediate **6** was reacted with different side chains (**12a–f**) in DMSO and heating at 80 °C for 8 h to obtain the first set of final target compounds **13a–f** that contains 3-methoxyphenyl at position 3 of pyrazole ring. The second set of final compounds **14a–f** was obtained by demthylation of methoxy group by using BBr_3_ in dichloromethane and cooling at 0 °C. The structures of final target compounds and their general structure are presented at Table 1.

**Table 1:** Structure of final target compounds 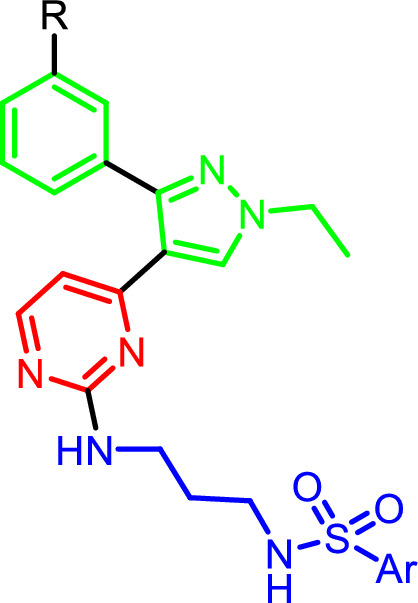

Comp No.	R	Ar
13a	OMe	
13b	OMe	
13c	OMe	
13d	OMe	
13e	OMe	
13f	OMe	
14a	OH	
14b	OH	
14c	OH	
14d	OH	
14e	OH	
14f	OH	

### Biological evaluation

#### Kinase inhibitory effect

The effect of all the synthesized pyrimidine-pyrazole hybrids **13a–f** and **14a–f** were tested in vitro on four kinase enzymes JNK1, JNK2, JNK3 and BRAFV600E. The IC_50_ (µM) values were calculated and compared to that of the lead compound **II** and the results are shown in Table 2. Compound **14c** exhibited the highest inhibitory activity on the four tested enzymes with IC_50_ = 0.51 μM, 0.53 μM, 1.02 μM and 0.009 μM on JNK1, JNK2, JNK3, and BRAFV600E, respectively. The activity of this compound was about 1.9 fold compared to the lead compound **II** on JNK enzymes. Interestingly, compound **14c** was more potent on BRAFV600E than the lead compound **II** by about 184 fold.

**Table 2:** IC_50_ of final target compounds over JNK1, JNK2, JNK3 and BRAFV600E

Comp No.	JNK1^a^	JNK2^a^	JNK3^a^	BRAFV600E^a^
13a	1.22 ± 0.08	1.75 ± 0.11	3.02 ± 0.14	0.77 ± 0.01
13b	1.03 ± 0.03	1.51 ± 0.23	2.66 ± 0.11	0.63 ± 0.02
13c	0.98 ± 0.07	1.30 ± 0.16	2.71 ± 0.22	0.57 ± 0.009
13d	0.99 ± 0.06	0.99 ± 0.04	2.33 ± 0.26	0.60 ± 0.03
13e	3.22 ± 0.18	2.98 ± 0.31	5.11 ± 0.61	1.96 ± 0.14
13f	5.66 ± 0.31	3.24 ± 0.17	7.22 ± 0.34	2.12 ± 0.26
14a	0.78 ± 0.020	0.79 ± 0.077	2.01 ± 0.31	0.042 ± 0.0011
14b	0.66 ± 0.014	0.61 ± 0.027	1.33 ± 0.17	0.035 ± 0.002
14c	0.51 ± 0.011	0.53 ± 0.013	1.02 ± 0.23	0.009 ± 0.0002
14d	0.55 ± 0.023	0.58 ± 0.022	1.31 ± 0.21	0.011 ± 0.0007
14e	1.88 ± 0.13	2.88 ± 0.21	4.31 ± 0.41	0.97 ± 0.13
14f	3.52 ± 0.22	4.22 ± 0.32	6.01 ± 0.72	1.33 ± 0.21
II	0.95 ± 0.09	1.01 ± 0.16	ND	1.66 ± 0.11

^a^Results are presented as mean of three experiments ± SD

#### Structure activity relationship

In this study the phenyl ring attached to the pyrazole was substituted with either methoxy (**13a–f**) or hydroxyl groups (**14a–f**) and also the aryl part of the benzesulfonamide moiety was decorated with electronically and hydrophobically different substituents to study their impact on the kinase inhibitory effect of the designed compounds.

Generally, the compounds with hydroxyl group **14a–f** showed higher activity over the four enzymes compared to their parent methoxy derivatives **13a–f**.

Regarding methoxy group containing series **13a–f**, compounds with electron withdrawing groups (**13b**, **13c**, and **13d**) showed higher activity over JNK isoforms and BRAFV600E compared to those bearing electron donating group (**13e**), bulky substituent (**13f**) or the unsubstituted derivative (**13a**). Compounds **13b–d** are equipotent on JNK1 and also similar in activity to the lead compound **II**. The activity of compounds **13b–d** was decreased by about 3–fivefold in their tolyl (**13e**) and naphthyl (**13f**) analogues. The same pattern was observed on JNK2 and JNK3 but with lower potencies compared to JNK1. Compound **13d** (p-F derivative) had the highest activity among this series with IC_50_ = 0.99 over JNK2 and 2.33 μM over JNK3 followed by compound **13b** (bromo derivative) with IC_50_ = 1.30 μM over JNK2 and 2.71 μM over JNK3. The naphthyl analogue **13f** was the least active compound on JNK2 and JNK3. Regarding BRAFV600E, all the compounds showed higher activity compared to JNK isoforms. Halogenated derivatives have similar potency over BRAFV600E with IC_50_ = 0.63 μM for **13b** (p-Br), 0.57 μM for **13c** (p-Cl) and 0.60 μM for **13d** (p-F). These compounds are more potent than the lead compound **II** by 2.6–2.9 fold. The unsubstituted compound **13a** is slightly less potent than its halogenated analogues with IC_50_ = 0.77 μM. Finally compounds **13e** (p-tolyl) and **13f** (naphthyl) have the lowest activity among this series with IC_50_ = 1.96 and 2.12 μM, respectively.

**Fig. 3:**
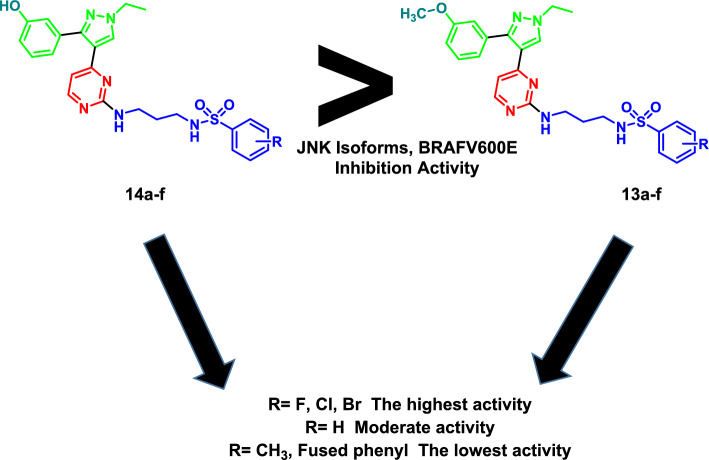
Structure activity relationship of the target pyrimidine-pyrazole hybrids against JNK isoforms and BRAFV600E

Concerning the second series which have hydroxyl group **14a–f**, compound **14c** (p-Cl) had the highest activity over the four enzymes with and it was more potent than its methoxy counterpart **13c** by about 2 and 63 fold on JNK isoforms and BRAFV600E, respectively. It was observed that both the p- bromophenyl **14b** and the p- fluorophenyl **14d** derivatives are slightly less potent than 14c on JNK isoforms. Meanwhile, on BRAFV600E the activity of compound **14b** is reduced by about threefold compared to **14c**. It is worth mentioning that the potency of the demethoxylated derivatives **14b** and **14d** are greater than their parent’s methoxy compounds **13b** and **13d** on BRAFV600E by about 18 and 54 fold, respectively. The unsubstituted derivative (**14a**) had moderate activity over the four enzymes and its activity was also higher than its analogue **13a** especially on BRAFV600E. Similarly as observed in the first series, the p-tolyl (**14e**) and naphthyl (**14f**) derivatives displayed the lowest activity in this group on JNK isoforms as well as BRAFV600E (Fig. 3).

#### Inhibitory effect on cancer cell lines

The second stage of biological evaluation was testing the final target compounds on different cancer cell lines, four cell lines were chosen based on the enzyme activity. MOLT-4 and K-562 represent leukemia cell lines and SK-MEL-28 and A375 represent melanoma cell lines.

In general, compounds **14a–f** showed higher activity compared to **13a–f**. Regarding MOLT-4 and K 562 cell lines, compound **14d** had the highest activity among all final target compounds with IC_50_ = 0.87 μM over MOLT-4 and 0.94 μM over K-562 followed by compound **14c** with IC_50_ = 0.94 and 0.99 μM over MOLT-4 and K-562. Compounds **14a, 14b, 14c**, and **14d** had higher activity compared to sorafenib over MOLT-4 with IC_50_ = 5.42 μM, 1.25 μM, 0.94 μM and 0.87, respectively. Similar manner was observed over K-562, both compounds **14c** and **14d** showed similar activity with IC_50_ 0.99 and 0.91 μM, respectively followed by compound **14b** with IC_50_ = 1.44 μM. Compounds **14a**, **14b, 14c**, and **14d** had a superior activity compared to sorafenib. Regarding SK-MEL-28 melanoma cell lines, compounds **13b**, **13c**, **13d**, **14a, 14b, 14c** and **14d** showed higher potency compared to sorafenib. Compound **14d** was the most potent with IC_50_ = 0.42 μM followed with compounds **14c** and **14b** with IC_50_ = 0.73 and 1.02 μM, respectively. The same was observed on methoxy containing compounds where compound **13d** was the highest potent compound with IC_50_ = 4.01 μM followed with compounds **13c** and **13b** with IC_50_ = 5.01 and 6.32 μM. On the other hand, compounds **13c, 13d, 14a, 14b**, **14c** and **14d** were more potent compared to sorafenib. The highest activity was achieved for compounds **14d** and **14c** with IC_50_ = 0.63 and 0.81 μM. It is worth to mention that bulky groups and electron donating groups showed the lowest activity compared to small and electron withdrawing groups (Table 3).

**Table 3:** IC_50_ of final target compounds over MOLT-4, K-562, SK-MEL-28 and A375 cell lines

Comp No.	MOLT-4	K-562	SK-MEL-28	A375
13a	11.24 ± 1.08	13.12 ± 1.21	10.97 ± 0.98	11.10 ± 0.51
13b	9.25 ± 0.51	9.54 ± 0.44	6.32 ± 0.81	7.21 ± 0.24
13c	8.54 ± 0.41	9.10 ± 0.71	5.01 ± 0.34	6.11 ± 0.71
13d	7.22 ± 0.45	8.11 ± 0.63	4.01 ± 0.12	4.89 ± 0.26
13e	18.34 ± 1.51	19.22 ± 1.01	12.57 ± 0.88	13.28 ± 1.39
13f	21.45 ± 1.88	26.86 ± 1.11	19.61 ± 1.34	20.11 ± 1.21
14a	5.42 ± 1.24	6.51 ± 0.24	2.41 ± 0.28	3.12 ± 0.77
14b	1.25 ± 0.08	1.44 ± 0.12	1.02 ± 0.04	1.11 ± 0.06
14c	0.94 ± 0.014	0.99 ± 0.012	0.73 ± 0.03	0.81 ± 0.02
14d	0.87 ± 0.011	0.91 ± 0.02	0.42 ± 0.01	0.63 ± 0.03
14e	11.41 ± 0.71	10.01 ± 0.18	8.21 ± 0.48	9.32 ± 0.36
14f	13.19 ± 1.05	10.56 ± 0.97	16.87 ± 1.36	18.52 ± 0.61
Sorafenib	6.12 ± 0.35	7.21 ± 0.28	5.22 ± 0.34	6.21 ± 0.25

Results are presented as mean of two experiments ± SD

#### Effect of compounds 14c and 14d on MEK1/2 and ERK1/2 phosphorylation

**Fig. 4:**
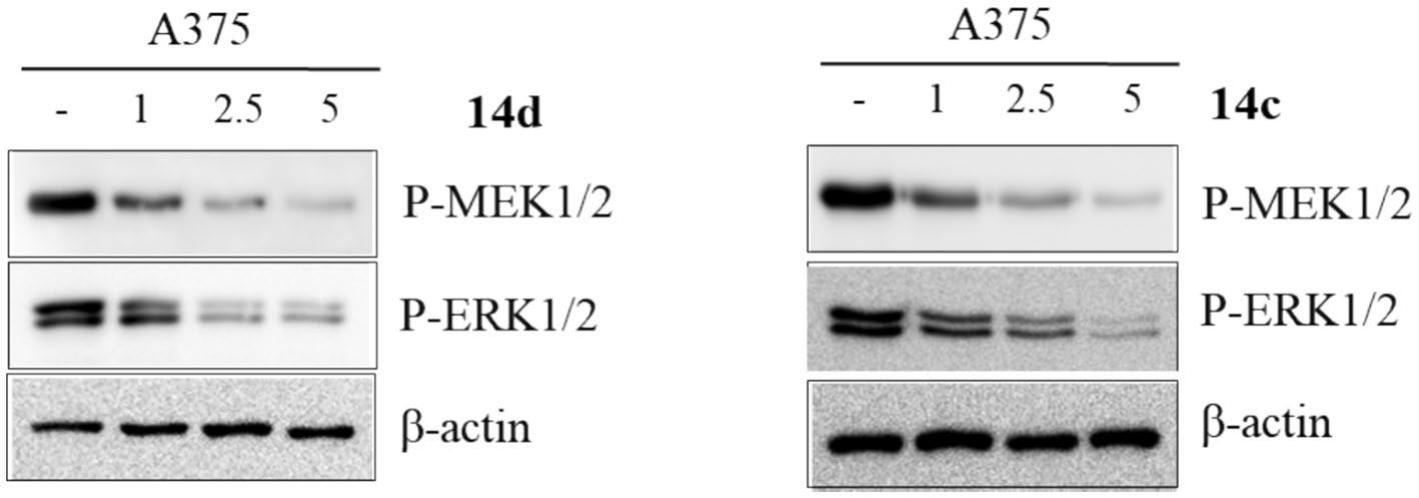
Western blot for compounds **14c** and **14d** on A375 cell line showing the effect of both compounds on MEK1/2 and ERK1/2 phosphorylation

Compounds **14c** and **14d** with the highest anticancer activities were testing using western blot to check the effect of BRAFV600E inhibition on phosphorylation of other MAPK pathway components. Both MEK1/2 and ERK1/2 were tested. Both tested compounds were used at three different concentrations 1, 2.5 and 5 μM. Compound **14c** showed significant inhibition for MEK phosphorylation starting from 2.5 μM and for ERK starting from 5 μM. Regarding compound **14d**, the significant inhibition of both MEK and ERK started from 2.5 μM with slight activity on MEK at 1 μM (Fig. 4).

#### Cell cycle analysis

**Fig. 5:**
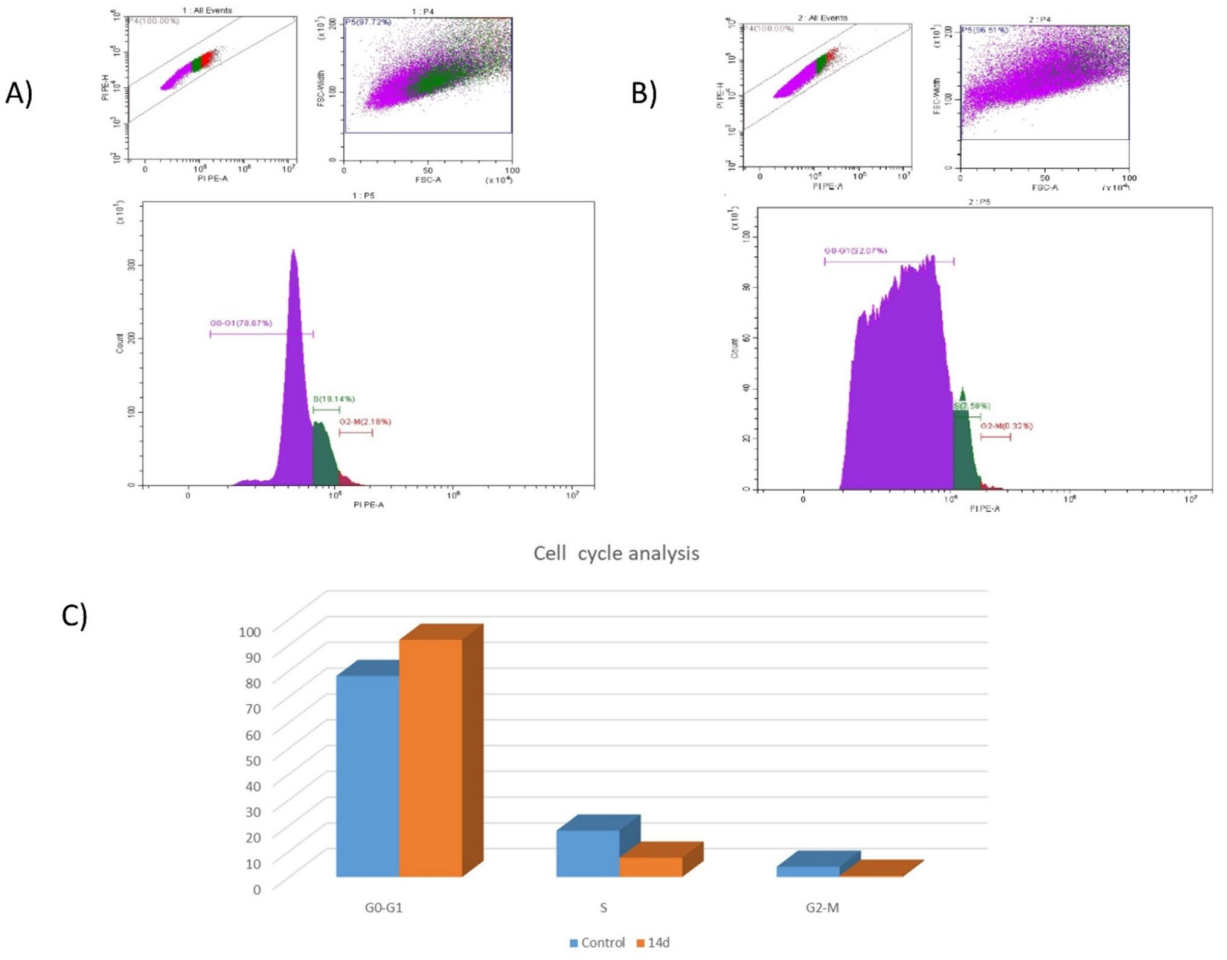
Effect of compound **14d** on cell cycle on A375 cancer cell line at 0.63 μM. **A** Control, **B** **14d**, **C** Percent of each phase in both control and tested compound **14d**

Compound **14d** that showed the highest anticancer activity was subjected to cell cycle analysis on A375 cell line at 0.63 μM (IC_50_) to detect the probable changes in the cell cycle phases in control and treated cells. The percentage of cells in G0/G1, S, and G2/M phases of the cell cycle was calculated using CytExpert Software. The treatment of A375 cells with sample **14d** showed an increase in the percentage of cells in the G0/G1 phase compared to negative control (from 78% in control group and 92% in tested compound)and decrease in both S phase and G2-M phase. The obtained results indicated that **14d** induced cell cycle arrest at the G0/G1 phase in the treated A375cells (Fig. 5).

#### Cell migration assay

**Fig. 6:**
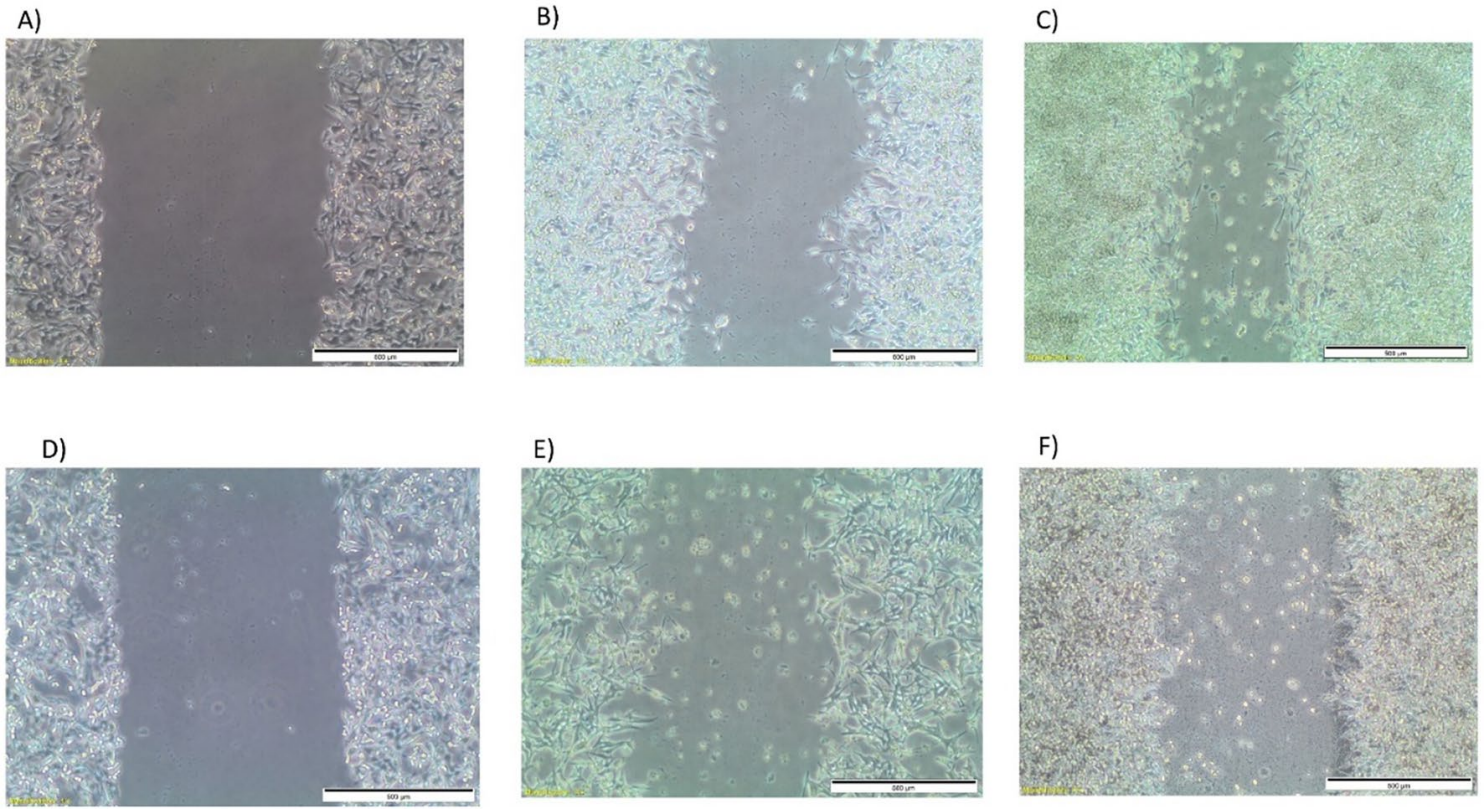
Migration test for compound **14d**; **A** control at 0 time; **B** control after 24 h; **C** control after 72 h; **D** compound **14d** at 0 time; **E** compound **14d** after 24 h; **F** compound **14d** after 72 h

One of the most important features of cancer cell is the ability to migrate from original location to other organs and accordingly causing metastasis. The migration test (pipet tip) is one of the common test to detect the ability of tested molecules to decrease or arrest cancer cell migration. In this assay, a monolayer of cells is scratched and exposure to a region devoid of cells causes the cells to migrate into the gap. The rate of cell migration is then observed under a microscope [48]. Compound **14d** was subjected to migration test at 0.63 μM (IC_50_) and it showed significant inhibition for cell migration starting from 24 h (Fig. 6).

#### Anti-inflammatory effect of final target compounds

As previously mentioned, JNK singling pathway way involved in inflammatory response. Therefore the final target compounds were screened for their anti-inflammatory effect by testing their ability to inhibit nitric oxide release and prostaglandin E2 production. Methoxy compounds **13a–f** showed high ability to inhibit nitric oxide release compared to hydroxyl compounds **14a–f** and compounds with halogen showed higher activity compared to unsubstituted and electron donating groups. Compounds **13b, 13c**, and **13d** showed more than 90% inhibition at 10 μM and had IC_50_ = 1.99 μM, 1.63 μM and 1.55 μM, respectively. Compounds **13a, 14b, 14c**, and **14d** showed more than 75% inhibition at 10 μM and showed IC_50_ = 8.66 μM, 2.89 μM, 3.97 μM, and 2.54 μM, respectively. On the other hand, final target compounds showed moderate PGE2 production inhibition, Compounds **14b, 14c** and **14d** showed more than 70% and IC_50_ = 8.02 μM, 7.58 μM, and 6.31 μM, respectively. Compounds **13b, 13c** and **13d** showed more than 50% inhibition for PGE2 production at 10 μM with IC_50_ = 9.18 μM, 9.65 μM and 10.21 μM. Unsubstituted derivatives **13a** and **14a** showed low inhibitory activity compare to compounds with electron withdrawing groups but higher compared to electron donating group such as **13e** and **14e**. Napthyl derivatives **13f** and **14f** did not show inhibitory activity at 10 μM (Table 4).

**Table 4:** Effect of final target compounds on nitric oxide release and PGE2 production on LPS induced RAW 264.7 macrophages

Comp No.	iNOS % Inhibition^a^	iNOS IC_50_^a^	PGE2% inhibition^a^	PGE2 IC_50_^a^
13a	75.61% ± 1.61	8.66 ± 0.19	21.52% ± 0.14	19.21 ± 0.42
13b	95.42% ± 1.34	1.99 ± 0.81	55.25% ± 1.01	9.18 ± 0.46
13c	97.57% ± 1.19	1.63 ± 0.07	59.31% ± 0.71	9.65 ± 0.22
13d	99.42% ± 1.21	1.55 ± 0.03	50.22% ± 0.32	10.21 ± 0.34
13e	67.51% ± 2.15	9.52 ± 0.51	12.31% ± 0.13	25.32 ± 0.82
13f	44.12% ± 2.18	13.2 ± 0.12	NA	NA
14a	60.32% ± 1.01	9.62 ± 0.71	41.29% ± 1.22	12.22 ± 0.31
14b	82.35% ± 1.61	2.89 ± 0.18	71.22% ± 1.31	8.02 ± 0.17
14c	79.59% ± 1.23	3.97 ± 0.70	75.31% ± 0.48	7.58 ± 0.21
14d	84.54% ± 1.88	2.54 ± 0.21	79.11% ± 0.21	6.31 ± 0.42
14e	32.82% ± 1.25	17.21 ± 0.58	29.32% ± 0.45	16.32 ± 0.81
14f	15.94% ± 1.02	20.19 ± 0.61	NA	NA

^a^Results are presented as mean of three experiments ± SD

#### Effect of compounds 13d and 14d on iNOS, COX-1 and COX-2

**Fig. 7:**
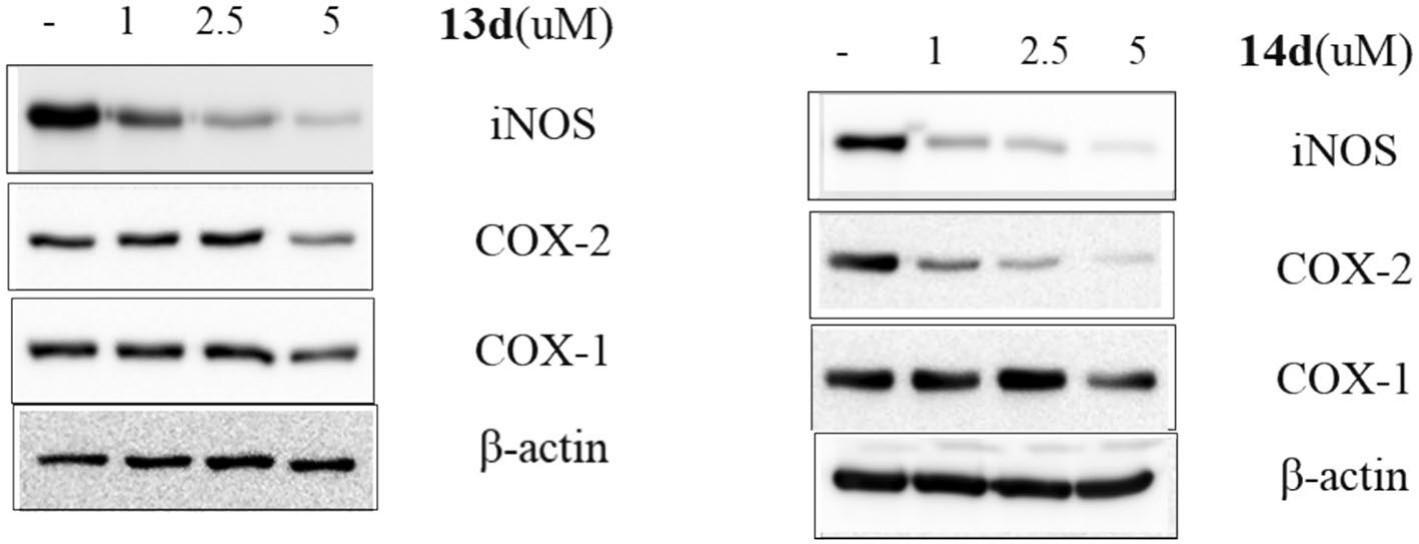
Western blot analysis of compound 1**3d** and **14d** over iNOS, COX-2 and COX-1

In addition to nitric oxide release and prostaglandin E2 production inhibition, the most potent compounds on pyrimidine series **13d** and **14d** were subjected to western blot analysis to check the effect on inducible nitric acid synthase, COX-1 and COX2 at three different doses 1, 2.5 and 5 μM. Compound **13d** showed remarkable decrease in iNOS starting from 2.5 μM and low effect on COX-2 at 5 μM and did not show any significant effect on COX-1 at tested doses. On the other hand, compound **14d** showed high effect on iNOS starting from 1 μM and significant effect on COX-2 starting from 2.5 μM and did not show any effect on COX-1 (Fig. 7).

#### Effect of final target compounds on cytokines production

**Fig. 8:**
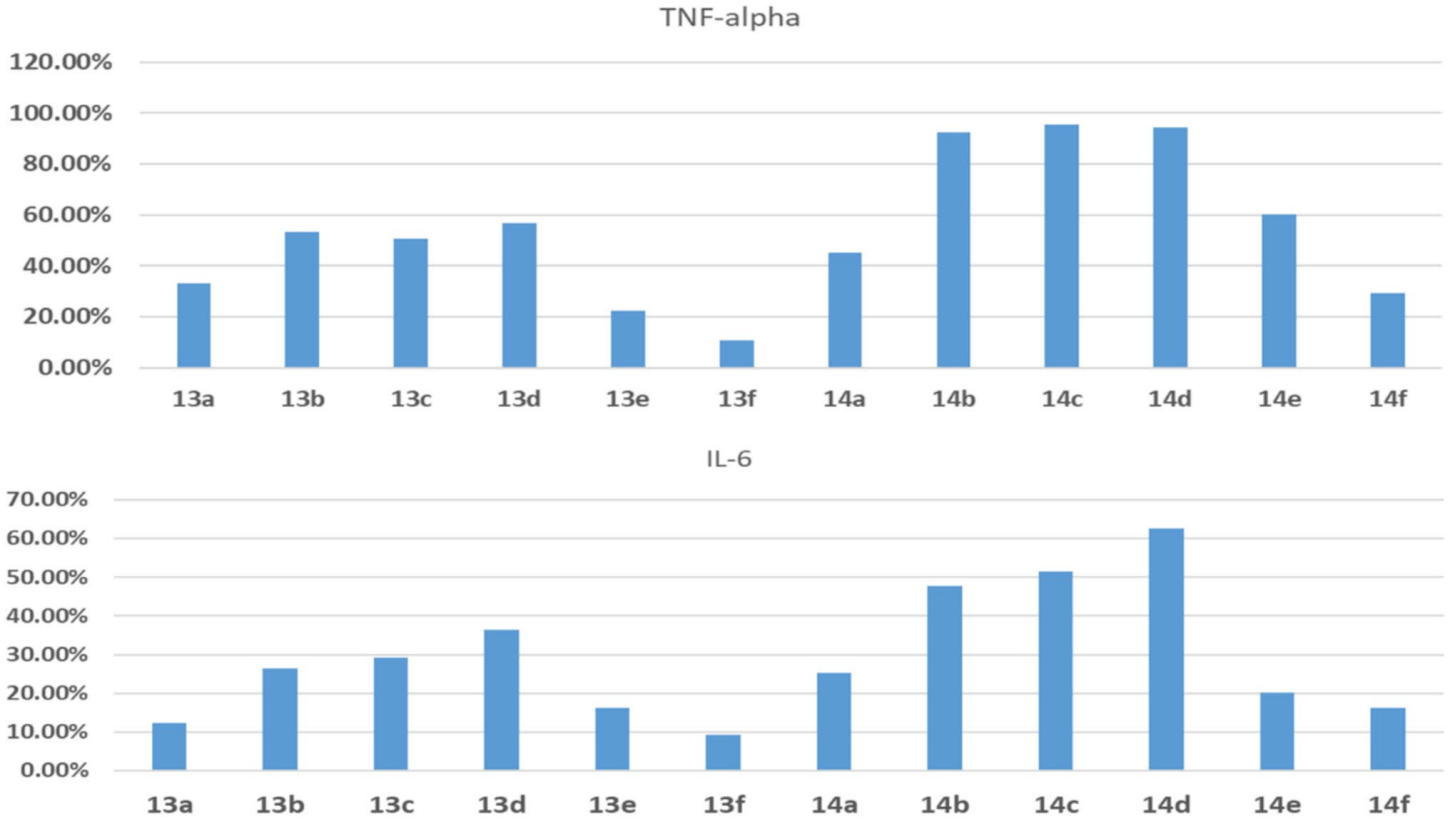
Effect of final target compounds on TNF-alpha, and IL-6 on LPS induced RAW 264.7 macrophages

Cytokines are a group of chemicals that participated in inflammatory process and main proteins that control inflammatory response to any stimuli. Tumor necrosis factor alfa, and interleukin-6 are considered the most important cytokines members. The final target compounds were tested at fixed dose 5 μM to detect their ability to inhibit production of TNF-alpha, and IL-6. TNF was inhibited by more than 80% under the effect of compounds **14b**, **14c**, and **14d** while compounds **13b, 13c, 13d, 14a**, and **14e** showed more than 40% inhibition at 5 μM. Regarding IL-6, compounds **14c** and **14d** showed more than 60% inhibition while compound **14b** showed more than 50% inhibition (Fig. 8; Table 5).

**Table 5:** IC_50_ of most potent final target compounds on TNF, IL-6 and their cell viability

Comp No.	TNF-alpha^a^	IL-6^a^	Cell viability^a^
14a	12.01 ± 0.11	33.21 ± 1.35	205 ± 2.11
14b	1.89 ± 0.05	11.21 ± 0.41	189 ± 1.92
14c	1.97 ± 0.14	9.35 ± 0.03	156 ± 3.14
14d	1.56 ± 0.06	7.22 ± 0.12	160 ± 2.33

^a^Results are presented as mean of three experiments ± SD

## Conclusion

In conclusion, two new series of pyrimidinyl ethyl pyrazoles derivatives **13a–f** and **14a–f** were designed and synthesized. The new compounds were designed based on the structure of reported strong BRAFV600E and JNKs inhibitor and using fragment based design; a new hybrid structure between the pyrimidine sulfonamide from BRAFV600E inhibitors and 3-substituted phenyl pyrazole from JNK inhibitors was produced. The new hybrid compounds showed good activity as anticancer and anti-inflammatory. Compounds with 3-hydroxyl phenyl at position 3 at the pyrazole ring **14a–f** were more potent compared to compounds having 3-methoxy phenyl **13a–f**. Also compounds with electron withdrawing groups were more potent compared to unsubstituted and compounds containing electron donating groups. Compound **14c** showed the highest activity on JNK1 and BRAFV600E with IC_50_ = 0.51 and 0.009 μM, respectively. Compounds **14b** and **14d** showed submicro-molar activity on JNK isoform and two digits nanomolar over BRAFV600E. Compounds **14c** and **14d** showed high activity over leukemia and melanoma cell lines. Compounds **14c** and **14d** inhibited the phosphorylation of both ERK1/2 and MEK1/2. Compound **14d** inhibited the cell cycle at G0-G1 phase and high ability to inhibit cell migration. Compounds **13-f** and **14a–f** exhibited moderate activity regarding nitric oxide release and PGE2 production and compounds **13d** and **14d** showed the highest activity in both tests. Compounds **13d** and **14d** were able to inhibit iNOS and COX-2 at low concentrations compared to COX-1. Compounds **14a–f** had a significant ability to inhibit the production of TNF-alpha and IL-6. Compounds **14a, 14b, 14c** and **14d** exhibited the higher ability to inhibit both TNF-alpha and IL-6. The new hybrid derivatives could be a lead compounds for development of new JNK and BRAFV600E inhibitors.

## Experimental

### Chemistry

Melting points were determined by the Electrothermal Capillary apparatus and were uncorrected. ^1^H NMR and ^13^C NMR spectra were measured on a Bruker Avance 400 or 300 and JEOL 400 spectrometers using TMS as an internal standard and chemical shift values were recorded in ppm ppm using the following abbreviation s (singlet), bs (broad singlet) d (doublet), dd (doublet of doublet), t (triplet), q (quartet), p (quintet), and m (multiplet). HRMS analysis was performed on a TIMS TOF- Bruker high-resolution mass spectrometer.

#### Synthesis of 1-(3-methoxyphenyl)-2-(2-(methylthio)pyrimidin-4-yl)ethan-1-one **(2)**

It was synthesized according to reported procedures in two step procedure [43].

##### Synthesis of methyl 3-methoxybenzoate **(1)**

To a solution of m-anisic acid (5.00 g, 32.9 mmol) in methanol (50 mL), sulfuric acid (1 mL) was added and the mixture was refluxed overnight. After reaction completion (TLC n-hexanes: ethyl acetate 6:1 Rf = 0.8), the reaction was cooled and excess methanol was removed. The residue was dissolved in ethyl acetate and washed with dist. water (3 × 100 mL) followed by aqueous sodium carbonate saturated solution (3 × 100 mL) and finally with dist. water (3 × 100 mL). The organic layer was separated, dried over anhydrous sodium sulfate and evaporated under reduced pressure to offer a pale yellow oily product (5.40 g, 99%).^1^H NMR (400 MHz, CDCl_3_) δ 7.61 (d, *J* = 6.12 *Hz*, 1H, Ar–H), 7.53 (s, 1H, Ar–H), 7.32 (s, 1H, Ar–H), 7.08 (d, *J* = 5.36 *Hz*, Ar–H), 3.89 (s, 3H, COOCH_3_), 3.82 (s, 3H, OCH_3_) [43].

##### Synthesis of 1-(3-methoxyphenyl)-2-(2-(methylthio)pyrimidin-4-yl)ethan-1-one **(2)**

To a solution of methyl 3-methoxybenzoate (**1**) (0.83 g, 5.0 mmol) and 4-methyl-2-(methylthio)pyrimidine (0.78 mL, 5.6 mmol) in THF (5 mL) in a cooled bath at − 25 °C, LHMDS (3.7 mL, 1.0 M solution in THF, 19.9 mmol) was slowly added at same temperature. After disappearing of ester on TLC, the mixture was concentrated. Ethyl acetate (3 × 25 mL) was added followed by water (100 mL). The combined organic layer extracts were washed with brine and dried over anhydrous sodium sulfate. The organic solvent was evaporated under reduced pressure and the residue was dissolved in methanol and crystalize TLC (Hexanes:Ethyl acetate 2:1), Rf: 0.8 to yield compound **2** (0.65 g, 49%), mp: 130–132 °C; ^1^H NMR (300 MHz, DMSO-*d*_*6*_) δ 14.38 (brs, 1H, OH, exchangeable), 8.58 (d, *J* = 4.48 *Hz*, 1H, Ar–H), 8.47 (d, *J* = 4.80 *Hz*, 1H, Ar–H), 7.63 (d, *J* = 7.28 *Hz*, 1H, Ar–H), 7.51(s, 1H, Ar–H), 7.47 (s, 1H, Ar–H), 7.46 (s, 1H, Ar–H), 7.39 (s, 2H, Ar–H), 7.24 (d, *J* = 7.96 *Hz*, 1H, Ar–H), 7.20 (d, *J* = 4.76 *Hz*, 1H, Ar–H), 7.07 (d, *J* = 7.52 *Hz*, 1H, Ar–H), 7.00 (d, *J* = 4.05 *Hz*, 1H, Ar–H), 6.45(s, 1H, Ar–H), 4.55 (s, 2H, CH_2_CO), 3.82 (s, 6H, OCH_3_), 2.58 (s, 3H, SCH_3_), 2.45 (s, 3H, SCH_3_).

#### Synthesis of 4-(3-(3-methoxyphenyl)-1H-pyrazol-4-yl)-2-(methylthio)pyrimidine **(4)**

A solution of compound **2** (9.00 g, 32 mmol) in DMF-DMA (82 mL) was stirred under reflux for 8 h. The reaction was monitored by TLC. The reaction was concentrated under vacuo and extracted with ethyl acetate (3 × 20 mL). The organic layer was dried in vacuo to give crude compound **3** and used for further reaction without purification. A mixture of compound **3** (12.00 g, 36 mmol.) and anhydrous hydrazine (1.6 mL, 50 mmol) in methanol (100 mL) was stirred under reflux for 18 h. Excess methanol was removed and the residue was partitioned between ethyl acetate (3 × 100 mL) and water (100 mL). The organic layer was separated and dried under vacuum to offer a pale yellow solid TLC (Hexanes:Ethyl acetate 2:1) Rf = 0.2, mp 131–133 °C, 61%, ^1^H NMR (400 MHz, CDCl_3_) δ 13.38 (brs, 1H, NH, exchangeable), 8.39 (d, *J* = 5.8 *Hz*, 1H, ArH), 8.28 (s, 1H, ArH), 7.32 (s, 1H, ArH), 7.07–7.04 (m, 4H, ArH), 3.73 (s, 3H, OCH_3_), 2.24 (s, 3H, CH_3_).^13^C NMR (100 MHz, DMSO-*d*_*6*_) δ 171.6 (Ar–C), 160.1 (Ar–C), 159.6 (Ar–C), 157.7 (Ar–C), 129.9 (Ar–C), 121.9 (Ar–C), 117.1 (Ar–C), 114.9 (Ar–C), 114.8 (Ar–C), 113.5 (Ar–C), 55.6 (OCH_3_), 13.5 (CH_3_).

#### Synthesis of 4-(1-ethyl-3-(3-methoxyphenyl)-1H-pyrazol-4-yl)-2-(methylsulfonyl) pyrimidine **(6)**

To a solution of compound **4** (5.00 g, 16 mmol) in DMF (30 mL) sodium hydride suspension (1.45 g, 35 mmol) was added with caution at 0 °C followed by ethyl iodide (2.80 mL, 35 mmol). The reaction mixture was stirred at room temperature for 12 h. Water (300 mL) was added dropwise. The product was obtained by extraction using ethyl acetate (3 × 150 mL). The organic layer was separated and dried under vacuum to give pale red residue of compound **5**. The residue was dissolved in anhydrous methanol (100 mL) and stirred at room temperature. A solution of oxone (16.50 g, 108 mmol) in H_2_O (200 mL) was added dropwise at room temperature. The reaction mixture was stirred at room temperature for 16 h. Methanol was removed under vacuum and the aqueous suspension was extracted with dichloromethane (3 × 150 mL). The organic layer was separated, dried over anhydrous sodium sulfate and evaporated. The residue was purified by column chromatography using silica gel and hexane:ethyl acetate 5:1 as mobile phase to give the titled compound as light red oil TLC (Hexanes:Ethyl acetate 2:1) Rf = 0.1, 45%, ^1^H NMR (400 MHz, CDCl_3_) δ 8.44 (d, *J* = 5.7 *Hz*, 1H, ArH), 8.15 (s, 1H, ArH), 7.18 (d, *J* = 7.6 *Hz*, 2H, ArH), 6.91(s, 2H, ArH), 6.82 (d, *J* = 7.6 *Hz*, 1H, ArH), 4.11 (d, *J* = 7.6 *Hz*, 2H, CH_2_ pyrazole), 3.65 (s, 3H, OCH_3_), 3.10 (s, 3H, CH_3_), 1.41 (t, *J* = 7.6 *Hz*, 3H, CH_3_ pyrazole).^13^C NMR (100 MHz, CDCl_3_) δ 165.9 (Ar–C), 161.3 (Ar–C), 159.7 (Ar–C), 157.9 (Ar–C), 151.2 (Ar–C), 134.3 (Ar–C), 132.4 (Ar–C), 129.8 (Ar–C), 121.3 (Ar–C), 119.3 (Ar–C), 116.7 (Ar–C), 114.6 (Ar–C), 114.3 (Ar–C), 55.3 (OCH_3_), 47.6 (CH_2_ pyrazole), 38.9 (CH_3_ pyrazole), 13.5 (CH_3_).

#### Synthesis of N-(3-aminopropyl)benzenesulfonamide derivatives **12a–f**

Side chains were synthesized according to reported procedures [41, 49].

##### Synthesis of benzyl (3-hydroxypropyl)carbamate **(7)**

To a stirred solution of 2-aminopropanol (6.12 mL, 81.86 mmol) in CH_2_Cl_2_ (50 mL) at 0 °C, TEA (22.20 mL, 159.6 mmol) was added. Benzyloxycarbonyl chloride (15.2 mL, 106.42 mmol) was added dropwise over 30 min. After completion of the addition, the mixture was stirred at 0 °C for 1 h. The mixture was quenched with water (50 mL) and extracted with CH_2_Cl_2_ (3 × 50 mL). The combined organic layer extracts were washed with brine, dried over anhydrous MgSO_4_, filtered, and concentrated under reduced pressure. The residue was purified by flash column chromatography. The desired product was obtained as white solid (12.33 g, 59%), TLC (hexanes:ethylacetate 5:1) Rf = 0.7. Colorless viscous oil; ^1^H-NMR (CDCl_3_, 300 MHz) δ 7.39 (s, 5H, ArH), 5.11 (bs, 1H), 5.07 (s, 2H, CH_2_ benzyl), 3.71 (s, 2H, OHCH_2_), 3.35 (q, 2H, *J* = 5.0 *Hz*, CH_2_NH), 1.72 (p, 2H,* J* = 5.0 *Hz*, CH_2_CH_2_CH_2_);^13^C NMR (CDCl_3_, 75 MHz) δ 157.2 (C = O), 136.4 (Ar–C), 128.5 (Ar–C), 128.1 (Ar–C), 128.0 (Ar–C), 66.8(CH_2_ benzyl), 61.7 (CH_2_OH), 43.4 (CH_2_NH), 29.2 (CH_2_CH_2_CH_2_); LC–MS: m/z calculated for C_11_H_15_NO_3_: 209.1 Found:210.3 (M + 1)^+^.

##### 3-(((Benzyloxy)carbonyl)amino)propyl methanesulfonate **(8)**

To a stirred solution of benzyl (2-hydroxypropyl)carbamate (**7**) (31.70 g, 152 mmol) in CH_2_Cl_2_ (300 mL) at 0°C, TEA (31.58 mL, 227 mmol) was added. Methanesulfonyl chloride (14.1 mL, 182 mmol) was then added dropwise to the reaction mixture over 30 min. After completion of the addition, the mixture was stirred at 0 °C for 1 h. The mixture was quenched with water (300 mL) and extracted with CH_2_Cl_2_ (3 × 300 mL). The combined organic layer extracts were washed with brine, dried over anhydrous sodium sulfate, filtered, and concentrated under reduced pressure. The residue was purified by flash column chromatography. The desired product was obtained (23.00 g, 53%) TLC (hexanes:ethylacetate 5:1) Rf = 0.4. As viscous oil that solidify with standing, mp 64–66°C; ^1^H-NMR (CDCl_3_, 300 MHz) δ 7.40 (s, 5H, ArH), 5.22 (bs, 1H), 5.09 (s, 2H, CH_2_-benzyl), 4.28 (t, 2H, *J* = 5.0 Hz, CH_3_SO_2_-CH_2_), 3.53 (q, 2H, *J* = 5.3 *Hz*, CH_2_-NH), 2.98 (s, 3H, CH_3_SO_2_), 1.70 (p, 2H, *J* = 5.3 *Hz*, CH_2_-CH_2_-CH_2_);^13^C NMR (CDCl_3_, 75 MHz) δ 156.4 (C = O), 136.3(Ar–C), 128.5 (Ar–C), 128.2 (Ar–C), 128.1 (Ar–C), 68.6 (CH_2_SO_2_CH_3_), 66.9(CH_2_ benzyl), 40.4 (CH_2_NH), 37.3 (CH_3_SO_2_), 28.9 (CH_2_CH_2_CH_2_); LC–MS: m/z calculated for C_12_H_17_NO_5_S: 287.08 Found:288.1 (M + 1)^+^.

##### Benzyl (3-azidopropyl)carbamate **(9)**

A mixture of sodium azide (4.75 g, 73.2 mmol) and 3-(((benzyloxy)carbonyl)amino)propyl methanesulfonate (**8**) (5.25 g, 18.3 mmol) in DMSO (50 mL) was stirred at 70°C for 2 h. The mixture was allowed to cool to room temperature, quenched with water (200 mL), and then extracted with ethyl acetate (3 × 200 mL). The combined organic layer extracts were washed with brine, dried over anhydrous sodium sulfate, filtered, and concentrated under reduced pressure. The title product was obtained as a colorless oil (3.90 g, 91%), TLC (hexanes:ethyl acetate 2:1) Rf = 0.8. ^1^H-NMR (CDCl_3_, 300 MHz) δ 7.34 (s, 5H, Ar–H), 5.11(s, 2H, Benzyl CH_2_), 3.41(d, 2H, *J* = 6.0 *Hz*, CH_2_NH), 3.35 (t, 2H, *J* = 4.5 *Hz*, CH_2_N_3_), 1.74 (p, *J* = 6.0 *Hz*, CH_2_CH_2_CH_2_);^13^C NMR (CDCl_3_, 75 MHz) δ 156.5 (C = O), 138.1 (Ar–C), 136.5 (Ar–C), 128.3 (Ar–C), 127.8 (Ar–C), 66.3 (CH_2_ benzyl), 40.5 (CH_2_NH), 40.2 (CH_2_N_3_), 30.1 (CH_2_CH_2_CH_2_); LC–MS: m/z calculated for C_11_H_14_N_4_O_2_: 234.11 Found:235.24 (M + 1)^+^.

##### Benzyl (3-aminopropyl)carbamate **(10)**

Triphenylphosphine (6.60 g, 25.5 mmol) and water (15 mL) were added to a solution of benzyl (3-azidopropyl)carbamate (**9**) (4.07 g, 17.4 mmol) in MeOH (40 mL). The mixture was heated under reflux for 2 h. The mixture was concentrated under reduced pressure and purified by column chromatography. TLC (Ethyl acetate:Methanol5:1) Rf = 0.3. The target product was obtained as light brown oil (3.25 g, 90%).^1^H-NMR (CDCl3, 300 MHz) δ 7.32 (s, 5H, Ar–H), 5.42 (bs, 1H,), 5.09 (s, 2H Benzyl CH_2_), 3.21 (q, 2H, *J* = 6.0 *Hz*, CH_2_NH), 2.78 (t, 2H,* J* = 6.0 *Hz*, CH_2_NH_2_), 1.72 (p, *J* = 6.0 *Hz*, 2H, CH_2_CH_2_CH_2_);^13^C NMR (CDC_l3_, 75 MHz) δ 156.8 (C = O), 136.6 (Ar–C), 128.5 (Ar–C), 128.0 (Ar–C), 66.6 (CH_2_ benzyl), 43.6 (CH_2_NH), 41.5 (CH_2_ NH2), 30.8 (CH_2_CH_2_CH_2_); LC–MS: m/z calculated for C_11_H_16_N_2_O_2_: 208.15 Found: 208.19 (M + 1)^+^.

##### General procedure for synthesis of benzyl (3-(phenylsulfonamido)propyl)carbamate derivatives **11a–f**

To a mixture of Benzyl (3-aminopropyl)carbamate (**10**) (0.16 g, 0.771 mmol), and TEA (0.32 mL, 2.32 mmol) in dry dichloromethane (20 mL), appropriate sulfonyl chloride (1.10 mmol) was added at 0 °C and the reaction mixture was stirred at room temperature for 12h (TLC Hexanes: ethyl acetate 1:2). The mixture was quenched with water (40 mL), and then extracted with ethyl acetate (3 × 40 mL). The combined organic layer extracts were washed with brine, dried over anhydrous sodium sulfate, filtered, and concentrated under reduced pressure. The residue was purified by flash column chromatography using hexanes:ethyl acetate 1:1.

*Benzyl (3-(phenylsulfonamido) propyl) carbamate* (**11a**) colourless oil, Rf = 0.6, 47%, ^1^H-NMR (CDCl3, 400 MHz) δ 7.71 (d, *J* = 7.62 *Hz*, 2H, Ar–H), 7.51 (t, *J* = 4.09 *Hz*, 1H, Ar–H), 7.33 (s, 5H, Ar–H), 6.95 (s, 1H Ar–H), 6.13 (s, 1H, Ar–H), 5.10 (s, 2H, CH_2_ benzyl), 3.59 (q, 2H, *J* = 5.3 *Hz*, CH_2_NHCO), 3.47 (q, 2H, *J* = 5.6 *Hz*, CH_2_NHSO), 1.73 (p, *J* = 5.5 *Hz*, CH_2_CH_2_CH_2_); LC–MS: m/z calculated for C_17_H_20_N_2_O_4_S: 348.11 Found:348.45 (M + 1)^+^.

*Benzyl (3-((4-bromophenyl)sulfonamido)propyl)carbamate* (**11b**), light brawn oil, Rf = 0.65, 59%, ^1^H-NMR (CDCl_3_, 300 MHz) δ 7.61–7.44 (m, 3H, Ar–H), 7.29 (s, 5H, Ar–H), 7.22–7.20 (m, 1H, Ar–H), 5.15 (s, 2H, CH_2_ benzyl), 3.41 (bs, 2H, CH_2_NHCO), 2.59 (t, *J* = 6.0 *Hz*, 2H, CH_2_NHSO), 1.75 (t, *J* = 6.0 *Hz*, 2H, CH_2_CH_2_CH_2_), LC–MS: m/z calculated for C_17_H_19_BrN_2_O_4_S: 426.02 Found:427.15 (M + 1)^+^.

*Benzyl (3-((4-chlorophenyl)sulfonamido)propyl)carbamate* (**11c**), viscous oil, Rf = 0.65, 64%, ^1^H-NMR (CDCl3, 300 MHz) δ 7.84 (d, *J* = 9.0 *Hz*, 2H, Ar–H), 7.35 (s, 5H, Ar–H), 7.59 (d, *J* = 9.0 *Hz*, 2H, Ar–H), 5.09 (s, 2H, CH_2_benzyl), 3.05 (t, *J* = 6.0 *Hz*, 2H, CH_2_NHCO), 2.81 (t, *J* = 6.0 *Hz*, 2H, CH_2_NHSO), 1.70 (p, *J* = 6.0 *Hz*, 2H, CH_2_CH_2_CH_2_). LC–MS: m/z calculated for C_17_H_19_ClN_2_O_4_S: 382.07 Found: 383.21(M + 1)^+^.

*Benzyl (3-((4-fluorophenyl)sulfonamido)propyl)carbamate* (**11d**), colorless oil, Rf = 0.55, 60%; ^1^H-NMR (CDCl_3_, 300 MHz) δ 7.80–7.76 (m, 2H, Ar–H), 7.28 (s, 5H, Ar–H), 7.11 (t, *J* = 6.0 *Hz*, 2H, Ar–H), 5.12 (s, 2H, CH_2_ benzyl), 2.89 (t, *J* = 6.0 *Hz*, 2H, CH_2_NHCO), 2.71(t, *J* = 6.0 *Hz*, 2H, CH_2_NHSO), 1.70 (p, *J* = 6.0 *Hz*, 2H, CH_2_CH_2_CH_2_). LC–MS: m/z calculated for C_17_H_19_FN_2_O_4_S: 366.10 Found: 367.21(M + 1)^+^.

*Benzyl (3-((4-methylphenyl)sulfonamido)propyl)carbamate* (**11e**), colorless oil, Rf = 0.7, 66%; 1H-NMR (CDCl3, 300 MHz) δ 7.78 (d, *J* = 9.0 *Hz*, 2H, ArH), 7.40 (d, *J* = 9.0 *Hz*, 2H, ArH), 7.22 (s, 5H, Ar–H), 5.08 (s, 2H, CH_2_benzyl), 2.86 (t, *J* = 6.0 *Hz*, 2H, CH_2_NHCO), 2.64 (t, *J* = 6 *Hz*, 2H, CH_2_NHSO), 2.42 (s, 3H, CH_3_); 1.72 (p, *J* = 6.0 *Hz*, 2H, CH_2_CH_2_CH_2_). LC–MS: m/z calculated for C_18_H_22_N_2_O_4_S: 362.13 Found: 363.21(M + 1)^+^.

*Benzyl (3-(naphthalene-1-sulfonamido)propyl)carbamate* (**11f**), White solid(71%); Rf = 0.8, mp.135–137 °C, ^1^H-NMR (CDCl_3_,400MHz) δ 8.01 (d, *J* = 8.0 Hz, 1H, Ar–H), 7.89–7.81 (m, 4H, Ar–H), 7.58 (d, *J* = 8.0 *Hz*, 2H, Ar–H), 7.34 (s, 5H), 5.14 (s, 2H, CH_2_benzyl) 3.06 (t, *J* = 6.0 *Hz*, 2H, CH_2_NHCO), 2.73 (t, *J* = 6 Hz, 2H, CH_2_NHSO), 1.66 (t, *J* = 6.4 *Hz*, 2H, CH_2_CH_2_CH_2_). LC–MS: m/z calculated for C_21_H_22_N_2_O_4_S: 398.13 Found: 399.21(M + 1)^+^.

##### General procedure for synthesis of N-(3-aminopropyl)benzenesulfonamide derivatives **12a–f**

10% Pd/C (0.04 g) was added to a solution of compound **11** (0.40 g, 1.25 mmol) in MeOH (20 mL). The mixture was stirred under hydrogen atmosphere at room temperature for 1 h. Pd/C was removed by celite filter, and the filtrate was evaporated under reduced pressure. and used in the next step without further purification (yield 80–91%).

#### General procedure for synthesis of final target compounds **13a–f**

A mixture of compound **6** (0.50 g, 1.3 mmol) with appropriate N-(3-aminopropyl)benzenesulfonamide (**12**) (4 mmol.) and DIPEA (1.7mL, 10 mmol.) in DMSO (5 mL) was heated at 80 °C for 16 h. (TLC Hexane:Ethyl acetate). The reaction was cooled and poured over ice and extracted with ethyl acetate (3 × 50 mL).The organic layer was dried over anhydrous sodium sulfate and evaporated under vacuum. The residue was purified using column chromatography (Hex:EA 2:1) to produce the desired final compounds.

*N-(3-((4-(1-Ethyl-3-(3-methoxyphenyl)-1H-pyrazol-4-yl)pyrimidin-2-yl)amino)propyl)benzenesulfonamide* (**13a**), white solid, Rf = 0.45, mp 122–124 °C, 68%, ^1^H NMR (400 MHz, CDCl_3_) δ 7.83 (s, 1H, ArH), 7.80 (d,* J* = 2.8 *Hz*, 1H, ArH), 7.77 (s, 1H, ArH), 7.52 (t,* J* = 9.6 *Hz*, 1H, ArH), 7.46–7.36 (m, 2H, ArH), 7.04 (d, *J* = 7.2 *Hz*, 1H, ArH), 6.97 (d, *J* = 7.6 *Hz*, 1H, ArH), 6.90 (s, 1H, ArH), 6.69 (s, 1H, NH, exchangeable), 6.32 (d, *J* = 9.6 *Hz*, 1H, ArH), 5.69 (s, 1H, ArH), 4.69 (s, 1H, NH, exchangeable), 4.27 (d, *J* = 2.0 *Hz*, 2H, CH_2_ pyrazole), 3.82 (s, 3H, OCH_3_), 3.21 (q, *J* = 5.6 *Hz*, 2H, NHCH_2_), 3.94 (s, 2H, CH_2_NH), 1.60 (q, *J* = 6.4 *Hz*, 2H, CH_2_CH_2_CH_2_), 1.51 (d, *J* = 2.4 *Hz*, 3H, CH_3_ pyrazole).^13^C NMR (100 MHz, CDCl_3_) δ 160.0(Ar–C), 158.3 (Ar–C), 145.8 (Ar–C), 143.8 (Ar–C), 140.6 (Ar–C), 136.2 (Ar–C), 134.6 (Ar–C), 132.8 (Ar–C), 130.6 (Ar–C), 129.2 (Ar–C), 127.8 (Ar–C), 124.2 (Ar–C), 120.2 (Ar–C), 117.9 (Ar–C), 115.9 (Ar–C), 112.1 (Ar–C), 105.4 (Ar–C), 55.0 (OCH_3_), 40.9 (CH_2_ pyrazole), 39.6 (NHCH_2_), 37.9 (CH_2_NH), 30.5 (CH_2_CH_2_CH_2_), 20.3 (CH_3_ pyrazole). HRMS calculated for C_25_H_28_N_6_O_3_S 492.1944, found 493.2022 [M + H]^+^.

*4-Bromo-N-(3-((4-(1-ethyl-3-(3-methoxyphenyl)-1H-pyrazol-4-yl)pyrimidin-2-yl)amino)propyl)benzenesulfonamide* (**13b**), pale red oil, Rf = 0.5, 65%, ^1^H NMR (400 MHz, CDCl_3_) δ 7.79 (d, *J* = 6.0 *Hz*, 1H, ArH), 7.75 (s, 1H, ArH), 7.58 (d, *J* = 7.2 *Hz*, 1H, ArH), 7.50 ( d, *J* = 8.2 *Hz*, 1H, ArH), 7.43–7.38 (m, 2H, ArH), 7.20 (t, *J* = 7.8 *Hz*, 1H, ArH), 7.03 (d, *J* = 8.4 *Hz*, 1H, ArH), 6.96 (d, *J* = 6.8 *Hz*, 1H, ArH), 6.88 (s, 1H, NH, exchangeable), 6.34 (d, *J* = 5.6 *Hz*, 1H, ArH), 5.98 (s, 1H, ArH), 4.76 (s, 1H, NH, exchangeable), 4.11 (d, *J* = 2.8 *Hz*, 2H, CH_2_ pyrazole), 3.82 (s, 3H, OCH_3_), 3.26 (brs, 2H, NHCH_2_) 2.96 (s, 2H, CH_2_NH), 1.64 (s, 2H, CH_2_CH_2_CH_2_), 1.51 (t, *J* = 2.8 *Hz*, 3H, CH_3_ pyrazole).^13^C NMR (100 MHz, CDCl_3_) δ 160.4 (Ar–C), 158.7 (Ar–C), 146.4 (Ar–C), 144.0 (Ar–C), 140.8 (Ar–C), 136.6 (Ar–C), 134.9 (Ar–C), 131.6 (Ar–C), 130.2 (Ar–C), 124.3 (Ar–C), 123.0 (Ar–C), 120.3 (Ar–C), 117.8 (Ar–C), 115.1 (Ar–C), 112.1 (Ar–C),55.9 (OCH_3_), 42.5 (CH_2_ pyrazole), 41.0 (NHCH_2_), 39.0 (CH_2_NH), 32.0 (CH_2_CH_2_CH_2_), 21.0 (CH_3_ pyrazole). HRMS calculated for C_25_H_27_BrN_6_O_3_S 570.1049, found 571.1127 [M + H]^+^.

*4-Chloro-N-(3-((4-(1-ethyl-3-(3-methoxyphenyl)-1H-pyrazol-4-yl)pyrimidin-2-yl)amino)propyl)benzenesulfonamide* (**13c**), white solid, Rf = 0.47, 112–114 °C, 69%, ^1^H NMR (400 MHz, CDCl_3_) δ 7.85 (d, *J* = 10.3 *Hz*, 1H, ArH), 7.77(s, 1H, ArH), 7.74 (s, 1H, ArH), 7.50–7.44 (m, 1H, ArH), 7.24–7.17 (m, 2H, ArH), 7.09 (d, *J* = 9.2 *Hz*, 1H, ArH), 6.93 (d, *J* = 10.4 *Hz*, 1H, ArH), 6.44 (s, 1H, NH, Exchangeable), 6.27 (dd,* J* = 5.6, 1.6 *Hz*, 1H, ArH), 5.95 (s, 1H, ArH), 4.59 (s, 1H, NH, exchangeable), 4.11 (d, *J* = 4.8 *Hz*, 2H, CH_2_ pyrazole), 3.82 (s, 3H, OCH_3_), 3.26 (q, *J* = 7.2 *Hz*, 2H, NHCH_2_), 2.96 (s, 2H, CH_2_NH), 1.64 (p, *J* = 5.6 *Hz*, 2H, CH_2_CH_2_CH_2_), 1.51 (t, *J* = 3.2 *Hz*, 3H, CH_3_ pyrazole).^13^C NMR (100 MHz, CDCl_3_) δ 163.0 (Ar–C), 159.0 (Ar–C), 147.0 (Ar–C), 142.7 (Ar–C), 139.2 (Ar–C), 136.1 (Ar–C), 132.3 (Ar–C), 130.6 (Ar–C), 129.4 (Ar–C), 127.4 (Ar–C), 120.8 (Ar–C), 118.9 (Ar–C), 116.6 (Ar–C), 114.2 (Ar–C), 111.9 (Ar–C), 55.4 (OCH_3_), 42.3 (CH_2_ pyrazole), 40.6 (NHCH_2_), 38.7 (CH_2_NH), 31.6 (CH_2_CH_2_CH_2_), 19.7 (CH_3_ pyrazole). HRMS calculated for C_25_H_27_ClN_6_O_3_S 526.1554, found 527.1632 [M + H]^+^.

*N-(3-((4-(1-Ethyl-3-(3-methoxyphenyl)-1H-pyrazol-4-yl)pyrimidin-2-yl)amino)propyl)-4-fluorobenzenesulfonamide* (**13d**), buff solid, mp 141–143 °C, Rf = 0.44, 74%, ^1^H NMR (400 MHz, CDCl_3_) δ 7.80 (d, *J* = 7.6 *Hz*, 1H, ArH), 7.77 (s, 1H, ArH), 7.59 (d, *J* = 7.6 *Hz*, 1H, ArH), 7.52 (d, *J* = 8.0 *Hz*, 1H, ArH), 7.42–4.37(m, 1H, ArH), 7.22 (d, *J* = 6.8 Hz, 1H), 7.14 (s, 1H, NH, exchangeable), 7.04 (d, *J* = 7.2 *Hz*, 1H, ArH), 6.96 (d, *J* = 6.8 *Hz*, 1H, ArH), 6.89 (s, 1H, ArH), 6.35 (d, *J* = 4.4 *Hz*, 1H, ArH), 6.00(s, 1H, ArH), 4.79 (s, 1H, NH, exchangeable), 4.09 (d, *J* = 2.8 *Hz*, 2H, CH_2_ pyrazole), 3.84 (s, 3H, OCH_3_), 3.25 (d, *J* = 7.2 *Hz*, 2H, NHCH_2_), 2.97 (s, 2H, CH_2_NH), 1.64 (t, *J* = 7.2 *Hz*, 2H, CH_2_CH_2_CH_2_), 1.51 (t, *J* = 1.6 *Hz*, 3H, CH_3_ pyrazole).^13^C NMR (100 MHz, CDCl_3_) δ 159.9 (Ar–C), 158.2 (Ar–C), 149.3 (Ar–C), 143.0 (Ar–C), 142.1 (Ar–C), 139.8 (Ar–C), 136.0 (Ar–C), 134.4 (Ar–C), 131.0 (Ar–C), 129.9 (Ar–C), 123.0 (Ar–C), 120.0 (Ar–C), 117.4(Ar–C), 115.0 (Ar–C), 111.9 (Ar–C), 105.3 (Ar–C), 55.4 (OCH_3_), 41.3 (CH_2_ pyrazole), 39.7 (NHCH_2_), 37.9 (CH_2_NH), 31.0 (CH_2_CH_2_CH_2_), 19.6 (CH_3_ pyrazole). HRMS calculated for C_25_H_27_FN_6_O_3_S 510.1849, found 511.1928 [M + H]^+^.

*N-(3-((4-(1-Ethyl-3-(3-methoxyphenyl)-1H-pyrazol-4-yl)pyrimidin-2-yl)amino)propyl)-4-methylbenzenesulfonamide* (**13e**), white solid, 113–115 °C, Rf = 0.6, 71%, ^1^H NMR (400 MHz, CDCl_3_) δ 7.80 (d, *J* = 7.2 *Hz*, 1H, ArH), 7.75 (s, 1H, ArH), 7.67 (d, *J* = 9.2 *Hz*, 1H, ArH), 7.38 (t, *J* = 6.8 *Hz*, 1H, ArH), 7.23 (d, *J* = 9.6 *Hz*, 2H, ArH), 7.03 (dd, *J* = 8.0, 2.8 *Hz*, 1H, ArH), 6.69 (d, *J* = 6.8 *Hz*, 1H, ArH), 6.45 (s, 1H, NH, exchangeable), 6.35 (dd, *J* = 5.6, 1.2 *Hz*, 1H, ArH), 5.98 (s, 1H, ArH), 4.68 (s, 1H, NH, exchangeable), 4.05 (d, *J* = 2.0 *Hz*, 2H, CH_2_ pyrazole), 3.83 (s, 3H, OCH_3_), 3.18 (q, *J* = 8.0 *Hz*, 2H, NHCH_2_), 2.94 (d,* J* = 6.0 Hz, 2H, CH_2_NH), 2.39 (s, 3H, CH_3_), 1.62 (q, *J* = 6.0 *Hz*, 2H, CH_2_CH_2_CH_2_), 1.51 (t, *J* = 2.8 *Hz*, 3H, CH_3_ pyrazole).^13^C NMR (100 MHz, CDCl_3_) δ 160.0 (Ar–C), 159.1 (Ar–C), 147.9 (Ar–C), 143.8 (Ar–C), 142.8 (Ar–C), 140.4 (Ar–C), 138.1 (Ar–C), 136.2 (Ar–C), 134.8 (Ar–C), 130.4 (Ar–C), 127.5 (Ar–C), 124.4 (Ar–C), 121.0 (Ar–C), 117.5 (Ar–C), 115.1 (Ar–C), 112.0 (Ar–C), 105.1 (Ar–C), 55.4 (OCH_3_), 42.0(CH_2_ pyrazole), 40.6(NHCH_2_), 39.0 (CH_2_NH), 31.3 (CH_2_CH_2_CH_2_), 29.5 (CH_3_), 19.6 (CH_3_ pyrazole). HRMS calculated for C_26_H_30_N_6_O_3_S 506.2100, found 507.2178 [M + H]^+^.

*N-(3-((4-(1-Ethyl-3-(3-methoxyphenyl)-1H-pyrazol-4-yl)pyrimidin-2-yl)amino)propyl)naphthalene-1-sulfonamide* (**13f**), white solid, Rf = 0.35, mp 78–80 °C, 62%, ^1^H NMR (400 MHz, CDCl_3_) δ 8.71 (d,* J* = 7.6 *Hz*, 1H, ArH), 8.22 (d, *J* = 7.6 *Hz*, 1H, ArH), 8.04 (d, *J* = 7.6 *Hz*, 1H, ArH), 7.92 (d, *J* = 8.4 *Hz*, 1H, ArH), 7.84 (d, *J* = 7.6 *Hz*, 1H, ArH), 7.60 (s, 1H, ArH), 7.58–7.48 (m, 2H, ArH), 7.38 (t, *J* = 8.4 *Hz*, 1H, ArH), 7.02 (dd, *J* = 8.4, 2.0 *Hz*, 1H, ArH), 6.95 (d, *J* = 7.6 *Hz*, 1H, ArH), 6.88 (s, 1H, ArH), 6.85 (brs, 1H, NH, exchangeable), 6.37 (d,* J* = 9.2 *Hz*, 1H, ArH), 5.96 (s, 1H, ArH), 4.67 (s, 1H, NH, exchangeable), 4.09 (d, *J* = 2.8 *Hz*, 2H, CH_2_ pyrazole), 3.83 (s, 3H, OCH_3_), 3.11 (t, *J* = 5.2 *Hz*, 2H, NHCH_2_), 2.95 (d,* J* = 6.0 *Hz*, 2H, CH_2_NH), 1.63 (t, *J* = 6.0 *Hz*, 2H, CH_2_CH_2_CH_2_), 1.51 (t, *J* = 4.0 *Hz*, 3H, CH_3_ pyrazole). ^13^C NMR (100 MHz, CDCl_3_) δ 160.6 (Ar–C), 159.0 (Ar–C), 148.3 (Ar–C), 143.2 (Ar–C), 140.6 (Ar–C), 136.9 (Ar–C), 134.6 (Ar–C), 134.0 (Ar–C), 129.6 (Ar–C), 129.1 (Ar–C), 128.9 (Ar–C), 128.1 (Ar–C), 126.7 (Ar–C), 124.7 (Ar–C), 124.1 (Ar–C), 123.7 (Ar–C), 120.9 (Ar–C), 118.3 (Ar–C), 115.5 (Ar–C), 112.4 (Ar–C), 105.7 (Ar–C), 55.7 (OCH_3_), 42.7 (CH_2_ pyrazole), 40.9 (NHCH_2_), 39.1 (CH_2_NH), 31.3 (CH_2_CH_2_CH_2_), 19.7 (CH_3_ pyrazole). HRMS calculated for C_29_H_30_N_6_O_3_S 542.2100, found 543.2178 [M + H]^+^.

#### General procedure for synthesis of final target compounds **14a–f**

The process of demethylation was performed using BBr_3_ according to reported procedure [50].

*N-(3-((4-(1-Ethyl-3-(3-hydroxyphenyl)-1H-pyrazol-4-yl)pyrimidin-2-yl)amino)propyl)benzenesulfonamide* (**14a**), white solid, TLC (Hexane: Ethyl acetate 1:3) Rf = 0.6, 132–134 °C, 32%, ^1^H NMR (400 MHz, MeOD) δ 7.84 (d, *J* = 8.8 *Hz*, 1H, ArH), 7.81 (s, 1H, ArH), 7.77 (d, *J* = 7.6 *Hz*, 1H, ArH), 7.60–7.50 (m, 2H, ArH), 7.32 (t, *J* = 7.6 *Hz*, 1H, ArH), 6.94 (dd, *J* = 8.4, 2.0 *Hz*, 1H, ArH), 6.84 (d, *J* = 8.4 *Hz*, 1H, ArH), 6.79 (s, 1H, ArH), 6.39 (dd, *J* = 5.6, 1.2 *Hz*, 1H, ArH), 6.10 (s, 1H, ArH), 4.24 (d, *J* = 2.8 *Hz*, 2H, CH_2_ pyrazole), 3.03 (t, *J* = 8.0 *Hz*, 2H, NHCH_2_), 2.89 (t,* J* = 7.6 *Hz*, 2H, CH_2_NH), 1.57 (t, *J* = 7.2 *Hz*, 2H, CH_2_CH_2_CH_2_), 1.56–1.49 (m, 3H, CH_3_pyrazole).^13^C NMR (100 MHz, MeOD) δ 159.3 (Ar–C), 158.1(Ar–C), 146.7 (Ar–C), 143.3 (Ar–C), 141.1 (Ar–C), 136.1 (Ar–C), 134.4 (Ar–C), 132.7 (Ar–C), 130.0 (Ar–C), 129.1 (Ar–C), 127.1 (Ar–C), 122.9 (Ar–C), 120.3 (Ar–C), 118.5 (Ar–C), 116.7 (Ar–C), 111.3 (Ar–C), 104.7 (Ar–C), 42.2 (CH_2_ pyrazole), 40.6 (NHCH_2_), 39.0 (CH_2_NH), 30.5 (CH_2_CH_2_CH_2_), 20.3 (CH_3_ pyrazole). HRMS calculated for C_24_H_26_N_6_O_3_S 478.1787, found 479.1865 [M + H]^+^.

*4-Bromo-N-(3-((4-(1-ethyl-3-(3-hydroxyphenyl)-1H-pyrazol-4-yl)pyrimidin-2-yl)amino)propyl)benzenesulfonamide* (**14b**), pale yellow solid, TLC (Hexane:Ethyl acetate 1:3) Rf = 0.55, 126–128 °C, 33%, ^1^H NMR (400 MHz, MeOD) δ 7.81 (s, 1H, ArH), 7.73–7.68 (m, 4H, ArH), 7.32 (t, *J* = 8.8 *Hz*, 1H, ArH), 6.95 (d, *J* = 7.6 *Hz*, 1H, ArH), 6.86 (d, *J* = 8.2 *Hz*, 1H, ArH), 6.80 (s, 1H, ArH), 6.41 (d, *J* = 7.6 *Hz*, 1H, ArH), 6.11 (s, 1H, ArH), 4.10 (d, *J* = 2.4 *Hz*, 2H, CH_2_ pyrazole), 3.02 (t, *J* = 8.0 *Hz*, 2H, NHCH_2_), 2.91 (t,* J* = 8.2 *Hz*, 2H, CH_2_NH), 1.57 (t, *J* = 7.2 *Hz*, 2H, CH_2_CH_2_CH_2_), 1.51 (t, *J* = 2.4 *Hz*, 3H, CH_3_ pyrazole).^13^C NMR (100 MHz, MeOD) δ 159.1 (Ar–C), 158.0 (Ar–C), 146.8 (Ar–C), 143.3 (Ar–C), 141.6 (Ar–C), 140.3 (Ar–C), 136.2 (Ar–C), 134.2 (Ar–C), 132.8 (Ar–C), 130.1 (Ar–C), 129.1 (Ar–C), 126.9 (Ar–C), 122.6 (Ar–C), 120.4 (Ar–C), 118.6 (Ar–C), 116.4 (Ar–C), 111.5 (Ar–C), 104.9 (Ar–C), 42.2 (CH_2_ pyrazole), 40.9 (NHCH_2_), 38.7 (CH_2_NH), 29.7 (CH_2_CH_2_CH_2_), 20.0 (CH_3_ pyrazole). HRMS calculated for C_24_H_25_BrN_6_O_3_S 556.0892, found 557.0970 [M + H]^+^.

*4-Chloro-N-(3-((4-(1-ethyl-3-(3-hydroxyphenyl)-1H-pyrazol-4-yl)pyrimidin-2-yl)amino)propyl)benzenesulfonamide* (**14c**), white solid, TLC (Hexane:Ethyl acetate 1:3) Rf = 0.5, mp 117–119 °C, 44%, ^1^H NMR (400 MHz, MeOD) δ 7.83–7.78 (m, 2H, ArH), 7.68 (d, *J* = 8.0 *Hz*, 1H, ArH), 7.52 (d, *J* = 9.2 *Hz*, 2H, ArH), 7.32 (t, *J* = 8.0 *Hz*, 1H, ArH), 6.95 (d, *J* = 8.2 *Hz*, 1H, ArH), 6.85 (d, *J* = 7.6 *Hz*, 1H, ArH), 6.80(s, 1H, ArH), 6.41 (dd,* J* = 5.6, 1.2 *Hz*, 1H, ArH), 6.13 (s, 1H, ArH), 4.10 (d, *J* = 0.8 *Hz*, 2H, CH_2_ pyrazole), 3.03 (t, *J* = 6.0 *Hz*, 2H, NHCH_2_), 2.91 (t,* J* = 7.2 *Hz*, 2H, CH_2_NH), 1.58 (q, *J* = 7.2 *Hz*, 2H, CH_2_CH_2_CH_2_), 1.50 (t, *J* = 1.2 *Hz*, 3H, CH_3_ pyrazole). ^13^C NMR (100 MHz, MeOD) δ 159.4 (Ar–C), 158.1 (Ar–C), 146.6 (Ar–C), 143.4 (Ar–C), 141.3 (Ar–C), 140.0 (Ar–C), 138.8 (Ar–C), 136.2 (Ar–C), 134.6 (Ar–C), 130.2 (Ar–C), 129.3 (Ar–C), 128.6 (Ar–C), 123.1 (Ar–C), 120.5 (Ar–C), 118.4 (Ar–C), 116.4 (Ar–C), 111.2 (Ar–C), 104.9 (Ar–C), 42.0 (CH_2_ pyrazole), 40.1 (NHCH_2_), 38.3 (CH_2_NH), 29.9 (CH_2_CH_2_CH_2_), 20.3 (CH_3_ pyrazole). HRMS calculated for C_24_H_25_ClN_6_O_3_S 512.1397, found 513.1476 [M + H]^+^.

*N-(3-((4-(1-Ethyl-3-(3-hydroxyphenyl)-1H-pyrazol-4-yl)pyrimidin-2-yl)amino)propyl)-4-fluorobenzenesulfonamide* (**14d**), white solid, TLC ( Hexane: Ethyl acetate 1:3) Rf = 0.55, mp 103–105 °C, 43%, ^1^H NMR (400 MHz, MeOD) δ 7.90 -7.87 (m, 1H, ArH), 7.87 (s, 1H, ArH), 7.69 (d, *J* = 6.8 *Hz*, 1H, ArH), 7.33 (t, *J* = 7.2 *Hz*, 1H, ArH), 7.24 (t, *J* = 8.8 *Hz*, 2H, ArH), 6.95 (dd, *J* = 8.4, 2.0 *Hz*, 1H, ArH), 6.87 (d, *J* = 7.6 *Hz*, 1H, ArH), 7.41 (dd, *J* = 5.6, 1.2 *Hz*, 1H, ArH), 6.16 (s, 1H, ArH), 4.09 (d, *J* = 1.6 *Hz*, 2H, CH_2_ pyrazole), 3.04 (t, *J* = 6.0 *Hz*, 2H, NHCH_2_), 2.90 (t,* J* = 5.8 *Hz*, 2H, CH_2_NH), 1.59 (t, *J* = 6.4 *Hz*, 2H, CH_2_CH_2_CH_2_), 1.51 (t, *J* = 1.6 *Hz*, 3H, CH_3_ pyrazole).^13^C NMR (100 MHz, MeOD) δ 160.1 (Ar–C), 157.9 (Ar–C), 147.9 (Ar–C), 143.9 (Ar–C), 141.9 (Ar–C), 138.6 (Ar–C), 136.9 (Ar–C), 136.2 (Ar–C), 134.9 (Ar–C), 130.7 (Ar–C), 130.7 (Ar–C), 124.1 (Ar–C), 120.7 (Ar–C), 119.4 (Ar–C), 116.7 (Ar–C), 111.5 (Ar–C), 105.7 (Ar–C), 42.3 (CH_2_ pyrazole), 40.7 (NHCH_2_), 36.8 (CH_2_NH), 30.3 (CH_2_CH_2_CH_2_), 19.1 (CH_3_ pyrazole). HRMS calculated for C_24_H_25_FN_6_O_3_S 496.1693, found 497.1771 [M + H]^+^.

*N-(3-((4-(1-Ethyl-3-(3-hydroxyphenyl)-1H-pyrazol-4-yl)pyrimidin-2-yl)amino)propyl)-4-methylbenzenesulfonamide* (**14e**), white solid, TLC ( Hexane: Ethyl acetate 1:3) Rf = 0.59, mp 132–134 °C, 41%, ^1^H NMR (400 MHz, MeOD) δ 7.81 (s, 1H, ArH), 7.70 (d, *J* = 6.8 *Hz*, 2H, ArH), 7.66 (d, *J* = 5.2 *Hz*, 1H, ArH), 7.35–7.31(m, 2H, ArH), 6.95 (dd, *J* = 8.4, 2.0 *Hz*, 1H, ArH), 6.85 (d, *J* = 7.6 *Hz*, 1H, ArH), 6.80 (s, 1H, ArH), 6.40 (dd, *J* = 5.6, 1.2 *Hz*, 1H, ArH), 6.15 (s, 1H, ArH), 4.13 (d, *J* = 2.0 *Hz*, 2H, CH_2_ pyrazole), 3.00 (t, *J* = 6.4 *Hz*, 2H, NHCH_2_), 2.87 (t,* J* = 7.6 *Hz*, 2H, CH_2_NH), 2.38 (s, 3H, CH_3_), 1.56 (t, *J* = 6.8 *Hz*, 2H, CH_2_CH_2_CH_2_), 1.49 (t, *J* = 1.6 *Hz*, 3H, CH_3_ pyrazole).^13^C NMR (100 MHz, MeOD) δ 160.0 (Ar–C), 158.1 (Ar–C), 146.6 (Ar–C), 143.8 (Ar–C), 142.9 (Ar–C), 141.1 (Ar–C), 137.5 (Ar–C), 135.9 (Ar–C), 134.3 (Ar–C), 129.5 (Ar–C), 129.3 (Ar–C), 126.6 (Ar–C), 122.4 (Ar–C), 120.3 (Ar–C), 118.6 (Ar–C), 116.6 (Ar–C), 105.1 (Ar–C), 43.5 (CH_2_ pyrazole), 41.0 (NHCH_2_), 39.0 (CH_2_NH), 30.6 (CH_2_CH_2_CH_2_), 24.2 (CH_3_), 20.2 (CH_3_ pyrazole). HRMS calculated for C_25_H_28_N_6_O_3_S 492.1944, found 493.2022 [M + H]^+^.

*N-(3-((4-(1-Ethyl-3-(3-hydroxyphenyl)-1H-pyrazol-4-yl)pyrimidin-2-yl)amino)propyl)naphthalene-1-sulfonamide* (**14f**), white solid, TLC (Hexane:Ethyl acetate 1:3) Rf = 0.4, mp 100–102 °C, 39%, ^1^H NMR (400 MHz, MeOD) δ 8.72 (d,* J* = 9.6 Hz, 1H, ArH), 7.20 (d, *J* = 8.4 *Hz*, 1H, ArH), 8.08 (d,* J* = 8.4 *Hz*, 1H, ArH), 7.96 (d, *J* = 8.4 *Hz*, 1H, ArH), 7.82 (s, 1H, ArH), 7.73–7.49 (m, 3H, ArH), 7.26 (t, *J* = 8.8 *Hz*, 1H, ArH), 6.91 (dd, *J* = 8.0, 2.0 *Hz*, 1H, ArH), 6.78–6.77 (m, 2H, ArH), 6.39 (dd, J = 5.6, 1.6 Hz, 1H, ArH), 6.01(s, 1H, ArH), 4.02 (d, *J* = 2.4 *Hz*, 2H, CH_2_ pyrazole), 2.90 (t, *J* = 7.6 *Hz*, 4H, NHCH_2_, CH_2_NH), 1.54 (t, *J* = 4.0 *Hz*, 2H, CH_2_CH_2_CH_2_), 1.49 (t, *J* = 1.2 *Hz*, 3H, CH_3_ pyrazole).^13^C NMR (100 MHz, MeOD) δ 159.2 (Ar–C), 157.9 (Ar–C), 146.3 (Ar–C), 143.2 (Ar–C), 141.1 (Ar–C), 135.8 (Ar–C), 135.4 (Ar–C), 134.5 (Ar–C), 134.1 (Ar–C), 133.7 (Ar–C), 129.7 (Ar–C), 128.9 (Ar–C), 128.5 (Ar–C), 128.2 (Ar–C), 127.8 (Ar–C), 126.8 (Ar–C), 124.7 (Ar–C), 124.1 (Ar–C), 122.6 (Ar–C), 120.2 (Ar–C), 118.0 (Ar–C), 116.6 (Ar–C), 110.8 (Ar–C), 104.3 (Ar–C), 43.7 (CH_2_ pyrazole), 40.5 (NHCH_2_), 38.7 (CH_2_NH), 30.4 (CH_2_CH_2_CH_2_), 23.6 (CH_3_ pyrazole). HRMS calculated for C_28_H_28_N_6_O_3_S 528.1944, found 529.2022 [M + H]^+^.

### Biological evaluation

The ethical approval of this study [Approval Number: 7445052023] is issued by the Medical Research Ethics Committee—National Research Centre—Egypt.

#### Enzyme assay

The enzymatic assays were performed in Reaction Biology Corp. using the standard protocol and at 1 μM ATP and threefold dilution factor [51].

#### Cellular activity

It was performed using MTT assay according to reported procedures [52].

#### Cell cycle

Cell cycle was performed for most potent compound using the reported procedures [53, 54].

#### NO determination

RAW 264.7 macrophages (1 × 10^5^ cells/ mL) were pre-incubated with sample for 60 min and then added with LPS (100 ng/mL) for one day. The supernatant was collected and nitrite levels in culture media were measured using Griess reaction and presumed to reflect nitric oxide levels [55].

#### Cell migration test

The test was performed on A375 cell line and using reported protocol [56].

#### Determination of PGE2, IL-6, and TNF-α production

RAW 264.7 macrophages (1 × 10^5^ cells/mL) were pre-incubated with sample for 1 h and then added with LPS (100 ng/mL) for 24 h. *N*-(2-Cyclohexyloxy-4-nitrophenyl)methanesulfonamide (NS398) (10 nM) was used as a positive control inhibitor.PGE2, TNF-α, and IL-6 levels in cell culture media were quantified by PGE2 (Enzo Life Sciences, Inc., Farmingdale, NY, USA) and TNF-α and IL-6 (BD Bio-science, Sand Diego, CA, USA) enzyme immunoassay (EIA) kits according to the manufacturer’s instructions [57].

#### Western blot

PRO-PREP™ (Intron Biotechnology, Seoul, Korea) supplemented with protease inhibitor cocktail (Sigma) was added to RAW 264.7 cells for protein extraction and these cells re-suspended in this reagent were kept for 30 min at 4 °C. Debris was separated using micro-centrifugation then the supernatants were frozen rapidly. The concentrations of the protein were measured following the manufacturer’s instruction for the Bio-Rad protein assay. Proteins (30 μg) were mixed with 5 × SDS sample buffer and boiled for 4 min then separated using electrophoresis on a 10% SDS-PAGE and electroblotted onto a PVDF membrane. The membrane was incubated for 60 min with blocking solution (5% skim milk) at 25 °C then kept overnight with a primary antibody at 4 °C (1:1000). Blots were washed 3 times with Tween20/Tris-buffered saline (T/TBS) and incubated with a 1:2000 dilution of HRP-conjugated secondary antibody for 2 h at 25 °C. Blots were again washed three times T/TBS then developed using an ECL chemiluminescence substrate (Santa Cruz, CA, USA). Autoradiographs were obtained by LAS-4000 luminescent image analyzer (Fujiflim, Tokyo, Japan). iNOS, COX-2, and β-actin antibodies were obtained from Santa Cruz Biotechnology Inc [57, 58].

## Supplementary Information

Below is the link to the electronic supplementary material.

Supplementary file1 (DOCX 5063 KB)

## Data Availability

No datasets were generated or analysed during the current study.
