# Supplementary material for: Design and synthesis of novel pyrimidine-pyrazole hybrids with dual anticancer and anti-inflammatory effects targeting BRAFV600E and JNK
Source: Mol Divers. 2025 Feb 22;29(6):6205–25. doi: 10.1007/s11030-025-11121-w (PMC12638350; doi:10.1007/s11030-025-11121-w)

**Design and synthesis of novel pyrimidine-pyrazole hybrids with dual anticancer and anti-inflammatory effects targeting V600EBRAF and JNK**

Mohammed S. Abdel-Maksoud,^a^***** Hebatollah E. Eitah^b^ _,_  Rasha M. Hassan^a^, Walaa Hamada Abd-Allah,^c^

*^a^Medicinal &Pharmaceutical Chemistry Department, Pharmaceutical and Drug Industries Research Institute, National Research Centre (NRC),(ID: 60014618), P.O. 12622, Dokki, Giza, Egypt .*

*^b^Medicinal &Pharmaceutical Chemistry Department, Pharmaceutical and Drug Industries Research Institute, National Research Centre (NRC),(ID: 60014618), P.O. 12622, Dokki, Giza, Egypt (Pharmacology Group).*

*^c^* *Pharmaceutical Chemistry Department, Collage of Pharmaceutical Science and Drug Manufacturing, Misr University for Science and Technology, P.O. 77, 6th of October City, Giza, Egypt*

*^*^* *Corresponding authors, Mohammed S. Abdel-Maksoud, Medicinal &Pharmaceutical Chemistry Department, Pharmaceutical and Drug Industries Research Institute, National Research Centre (NRC),(ID: 60014618), P.O. 12622, Dokki, Giza, Egypt .*

*E-mail:* [*Ph_ss@hotmail.com*](mailto:Ph_ss@hotmail.com)

**
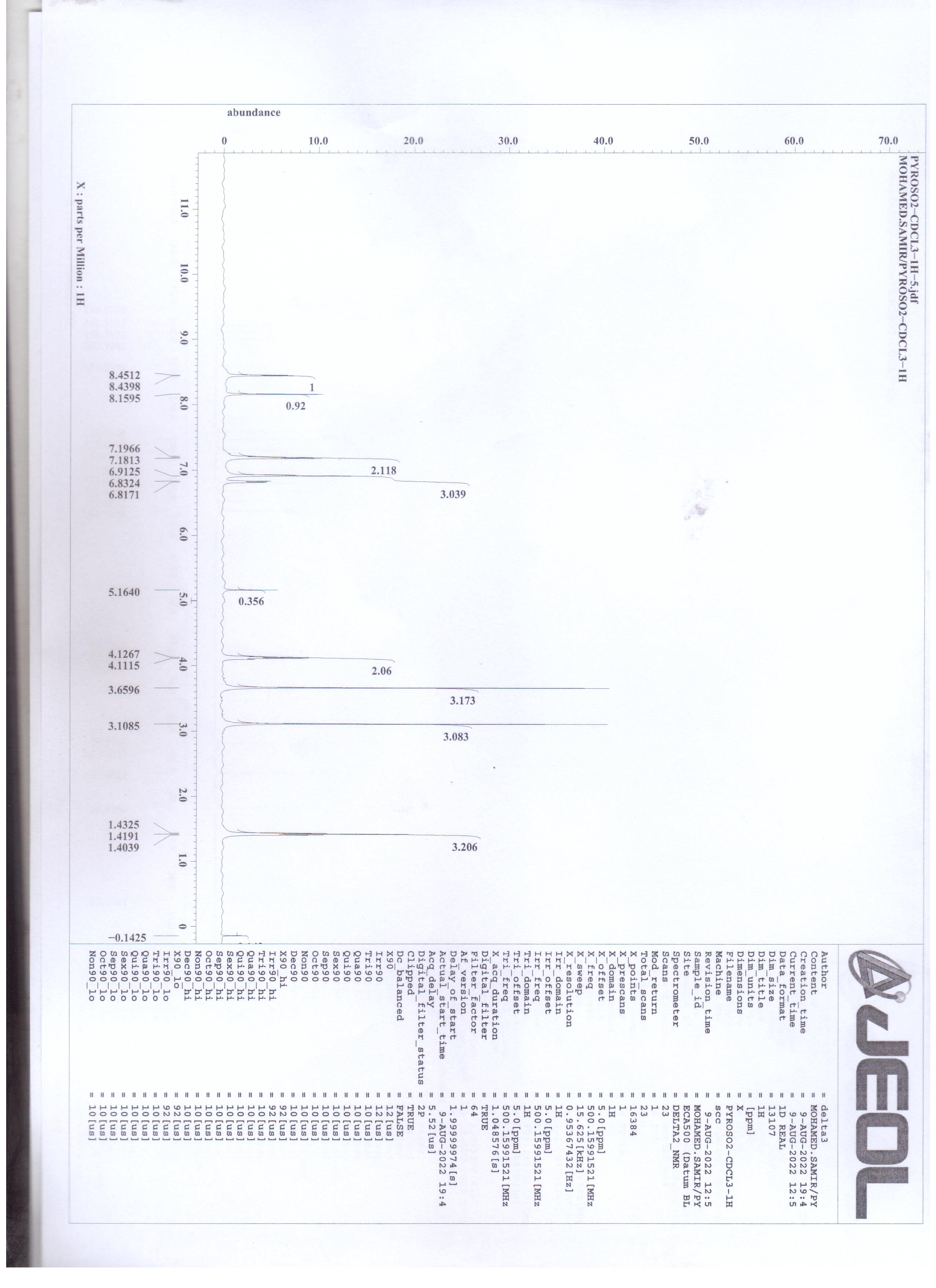
**

**
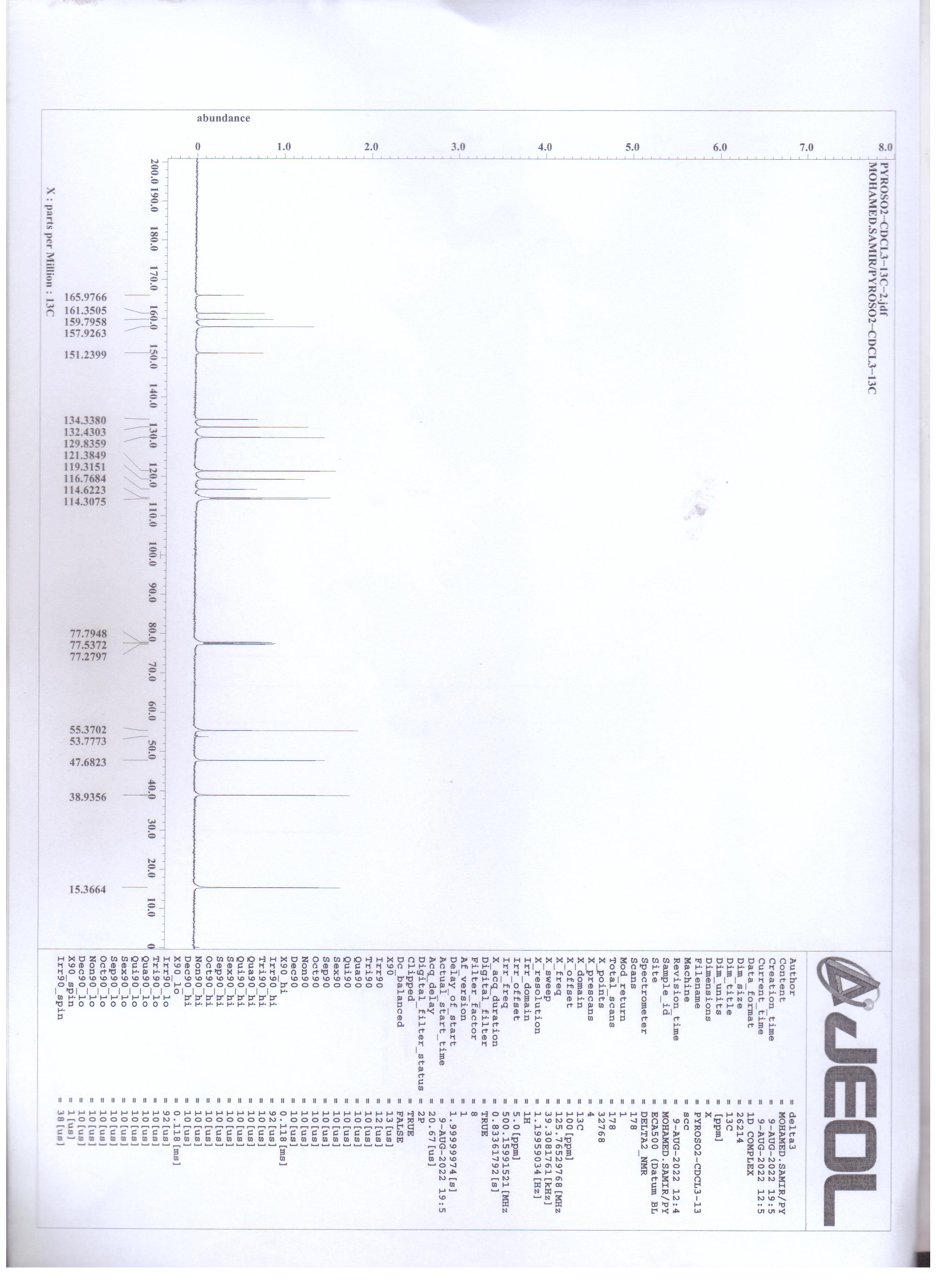
**

**13a**


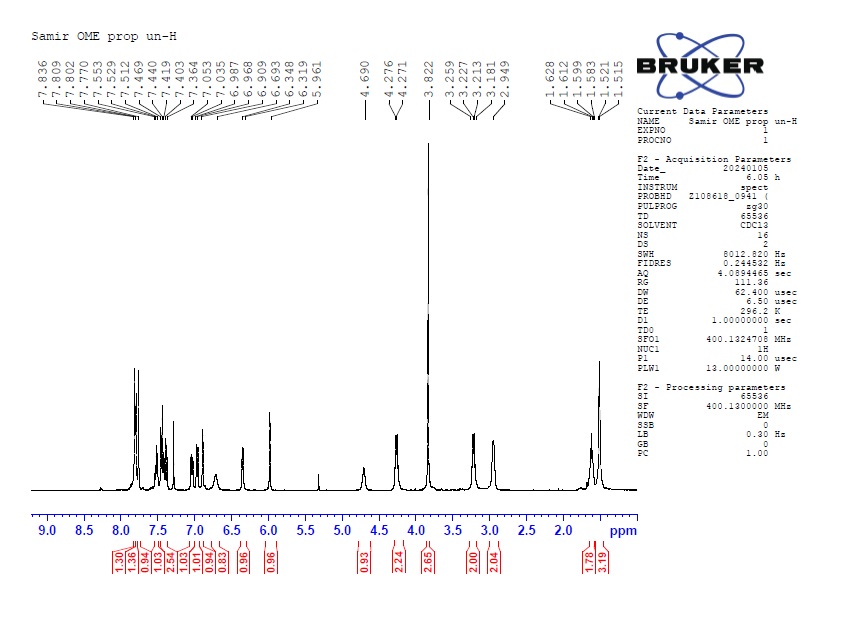

**13a**


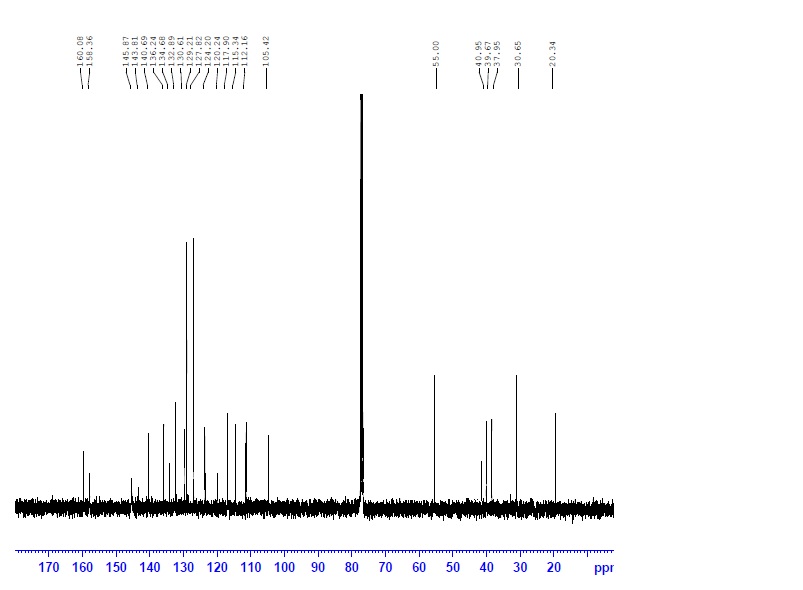

**13a**


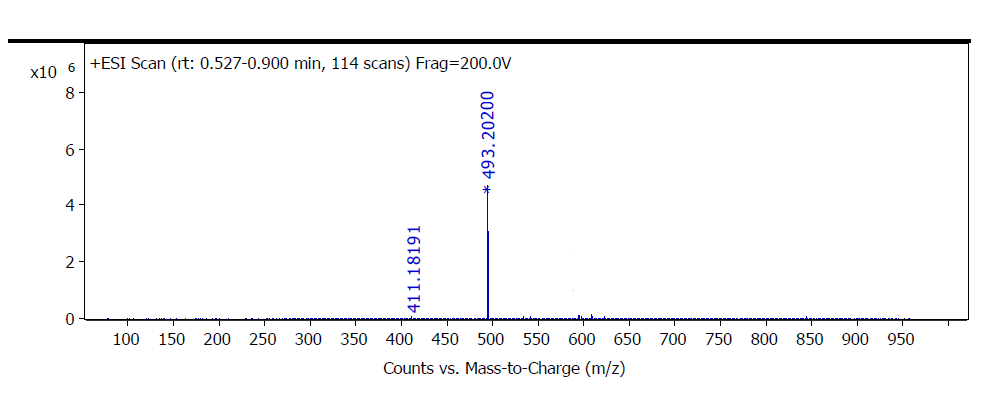

**13b**


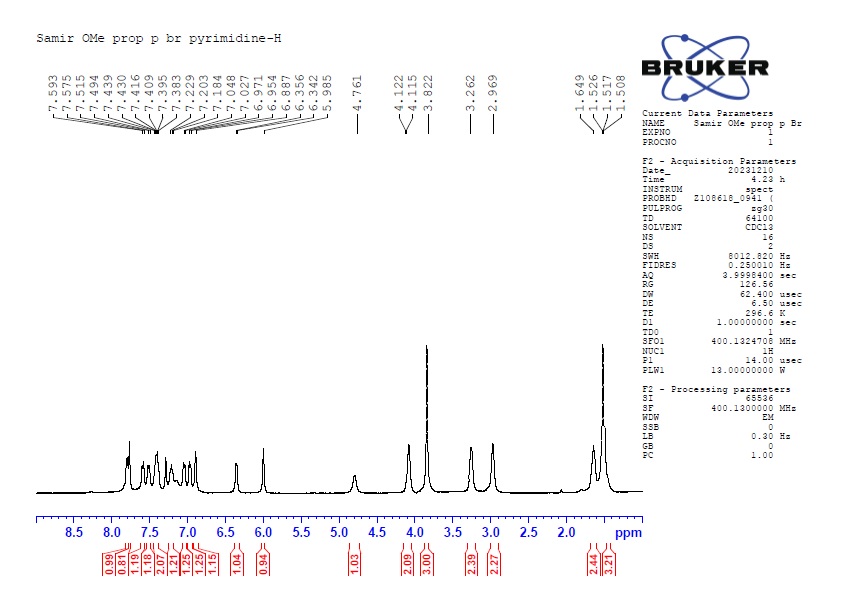

**13b**

**13b**


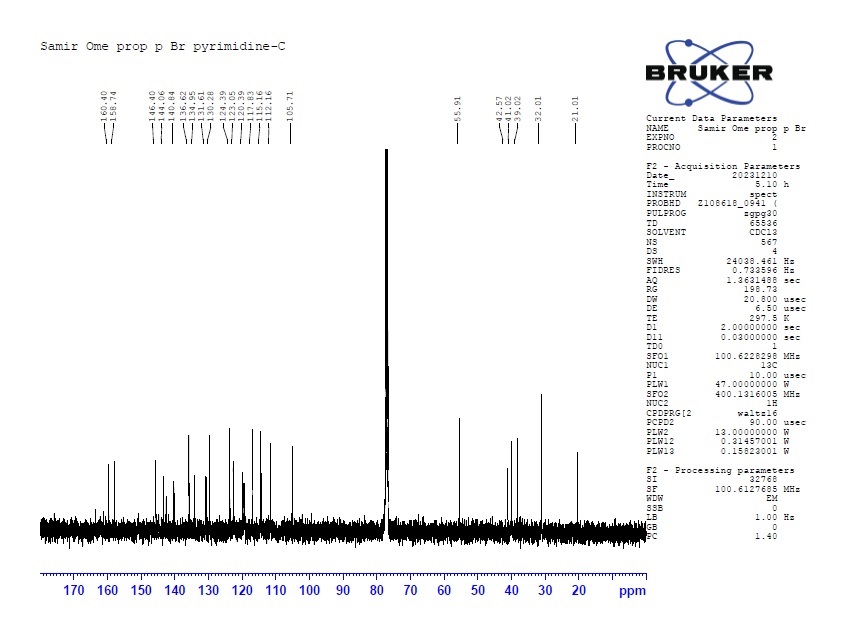

**13b**


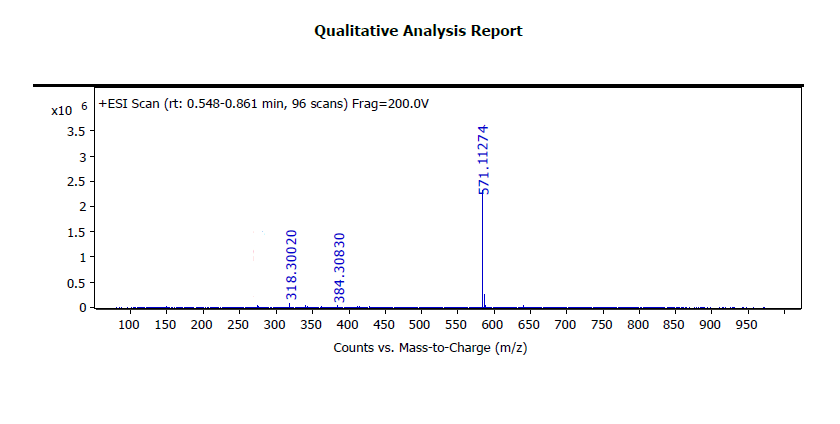

**13c**


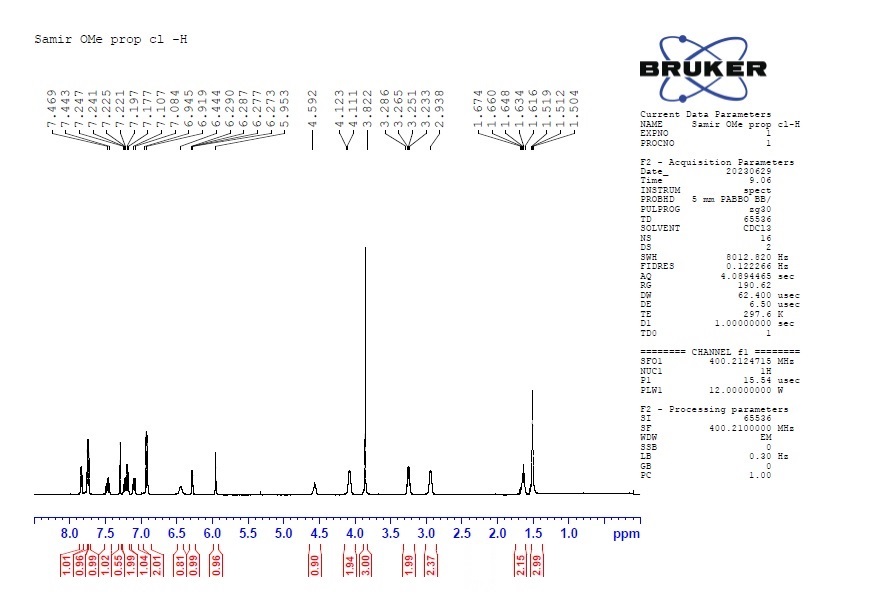

**13c**


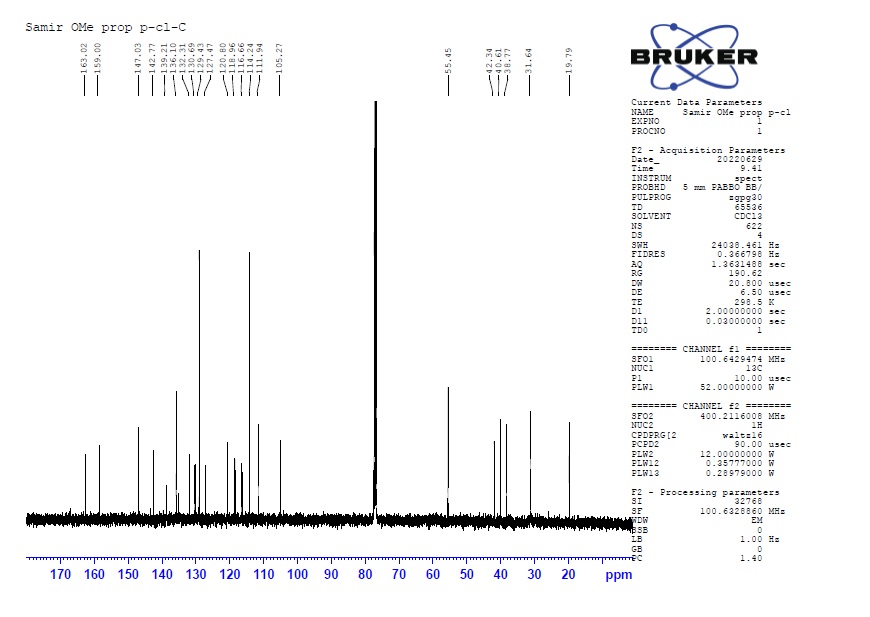

**13c**


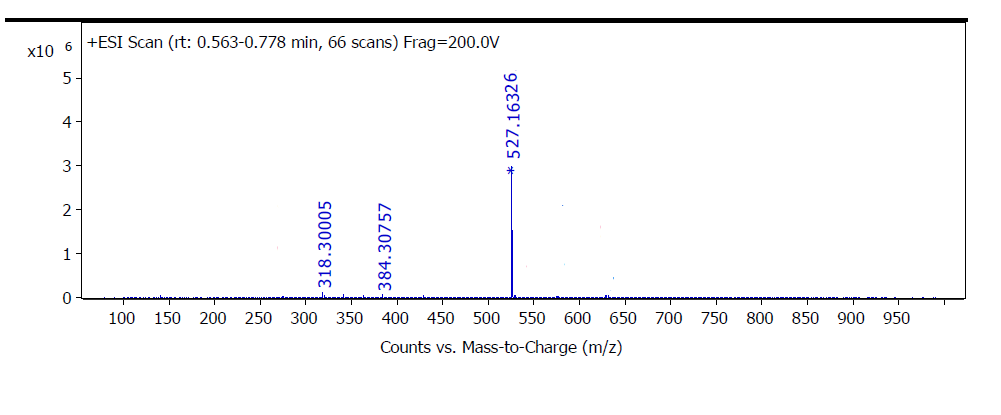

**13d**


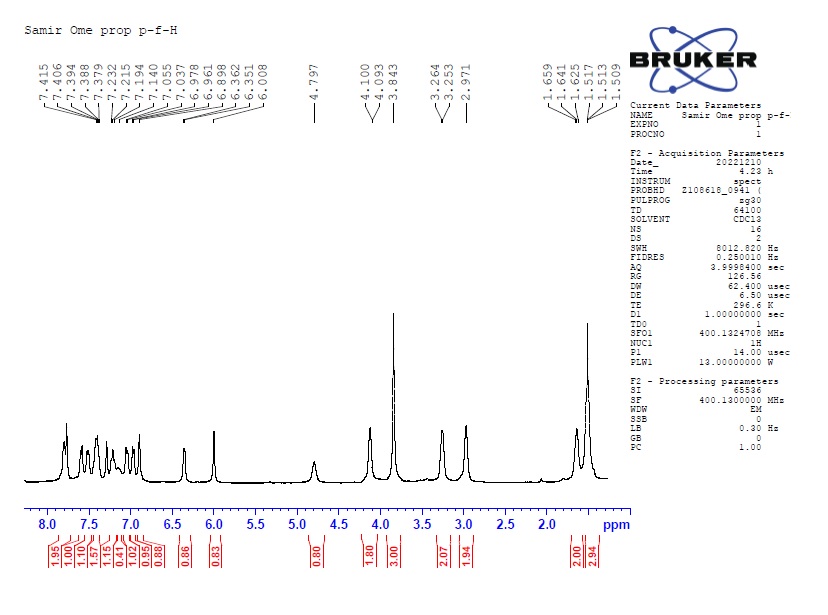

**13d**


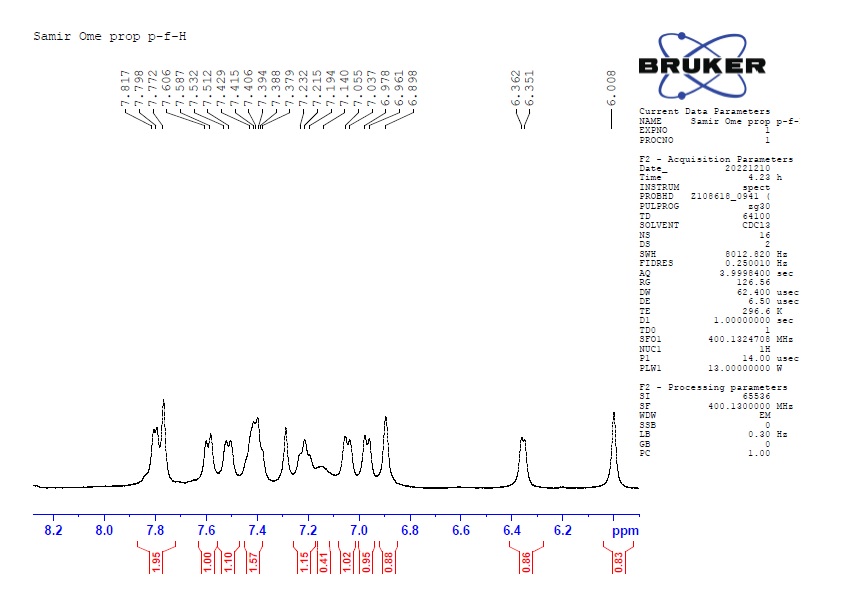

**13d**


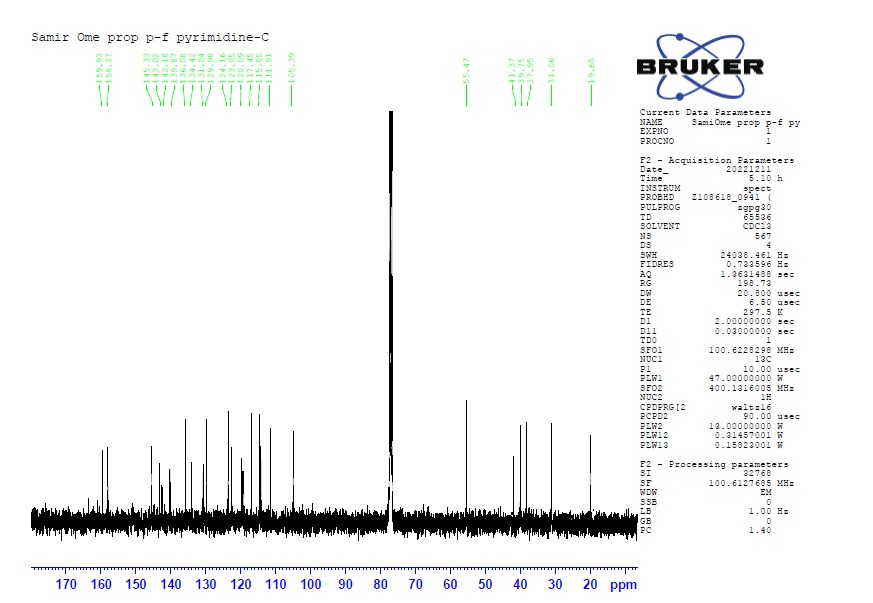

**13d**


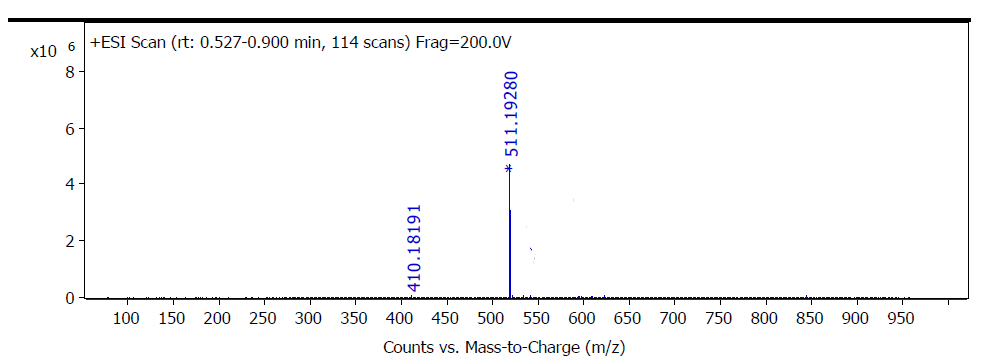

**13e**


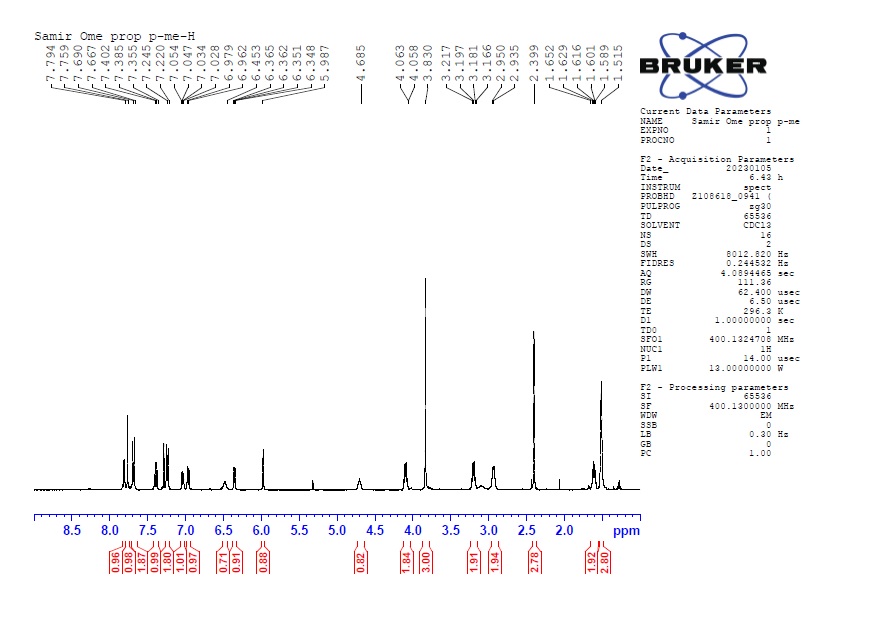

**13e**


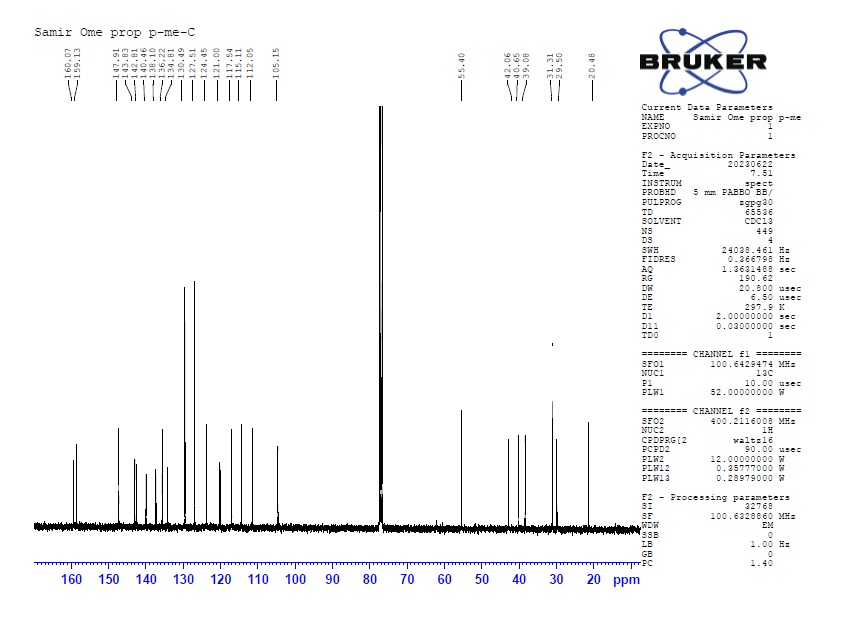

**13f**


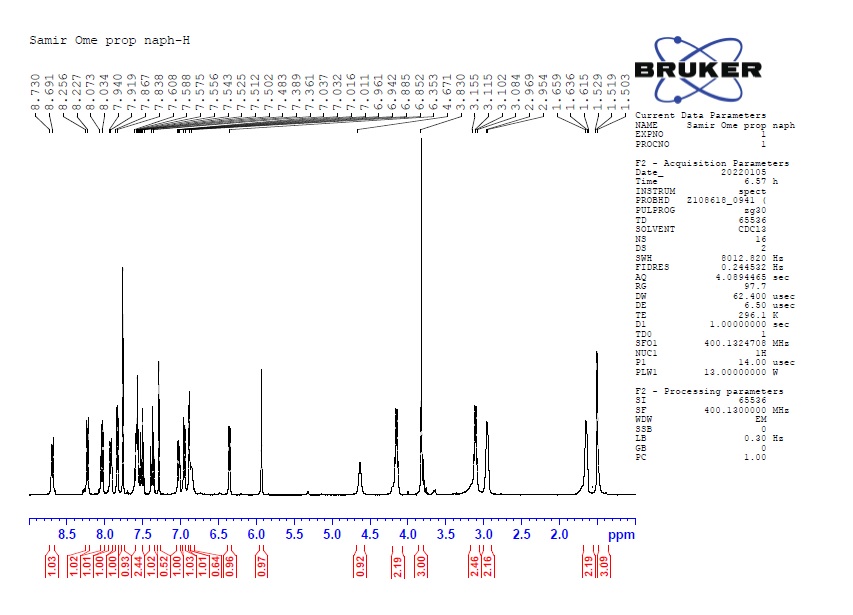

**13f**


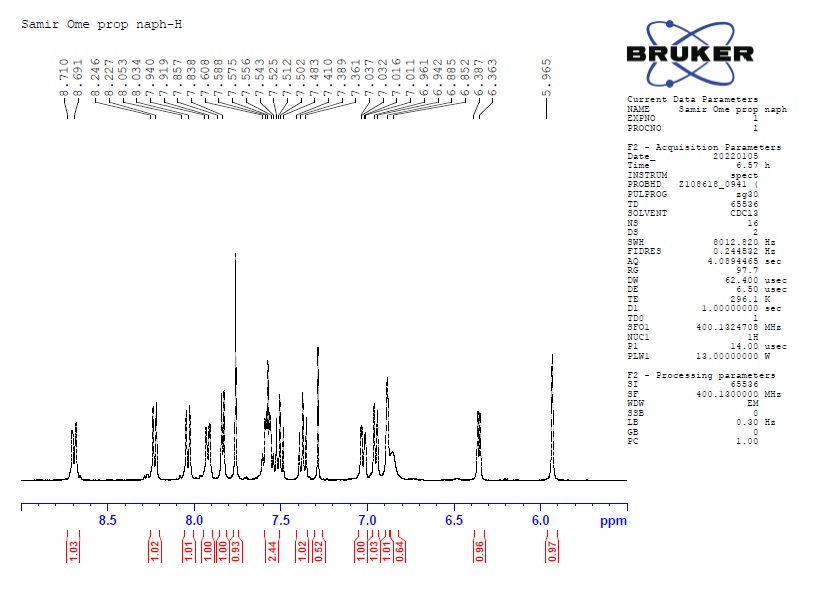

**13f**


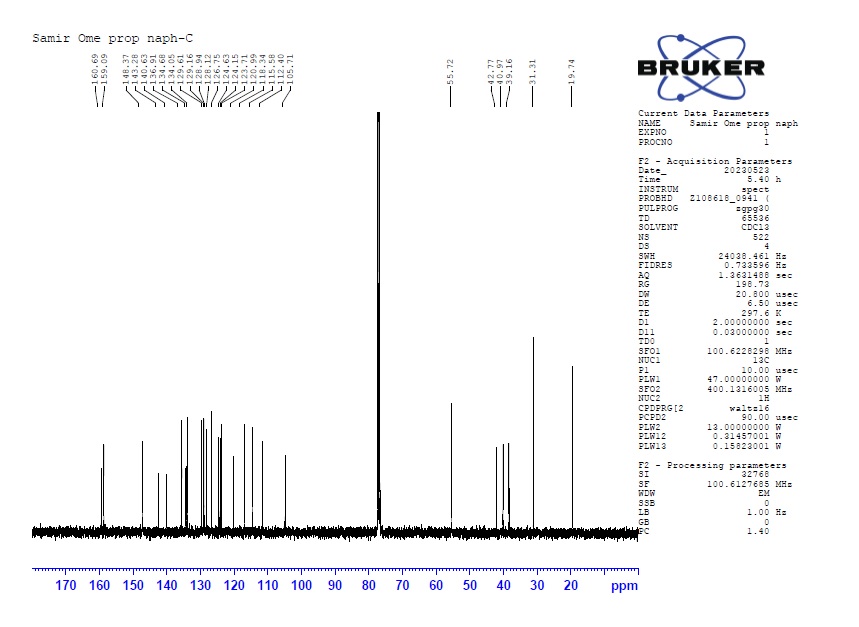

**14a**


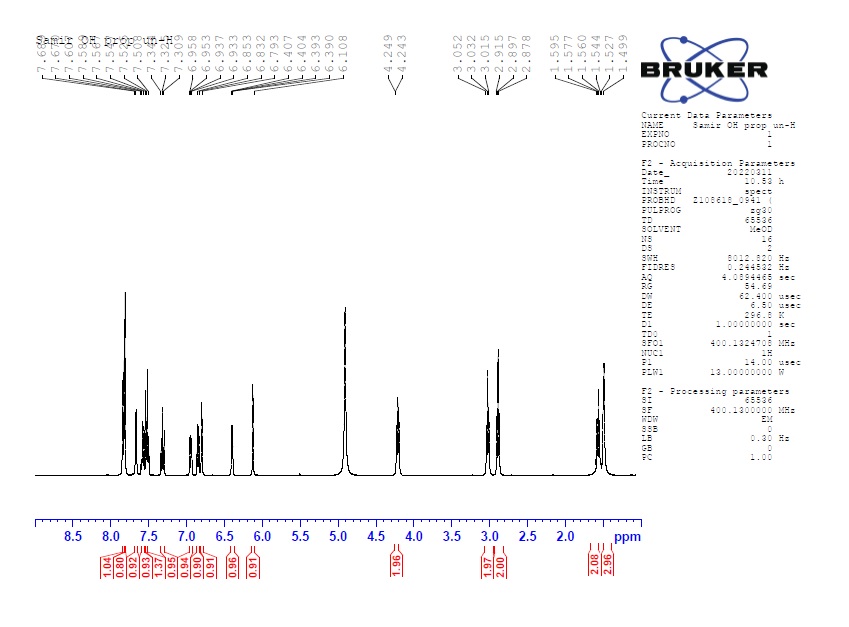

**14a**

**14a**


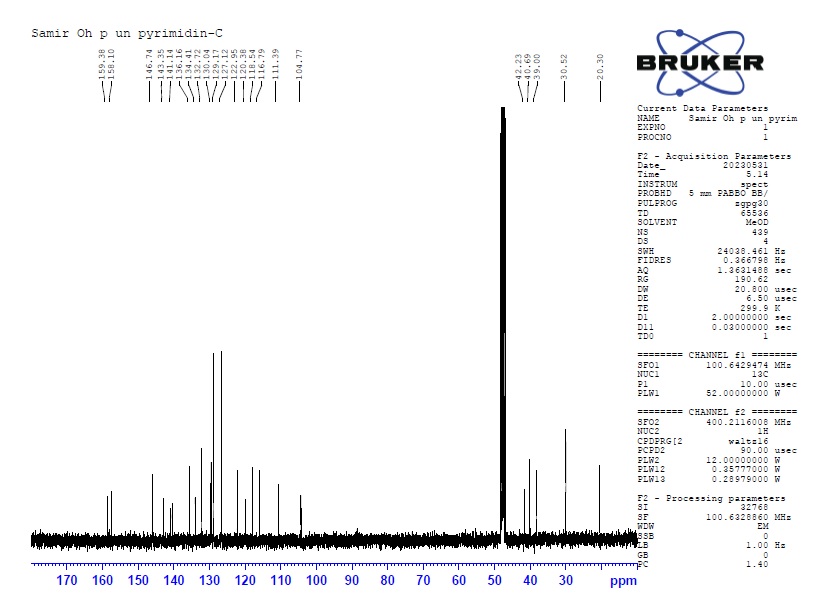

14a


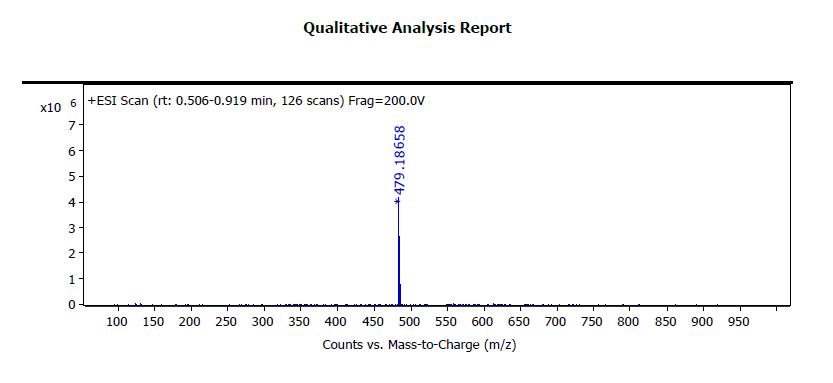

**14b**


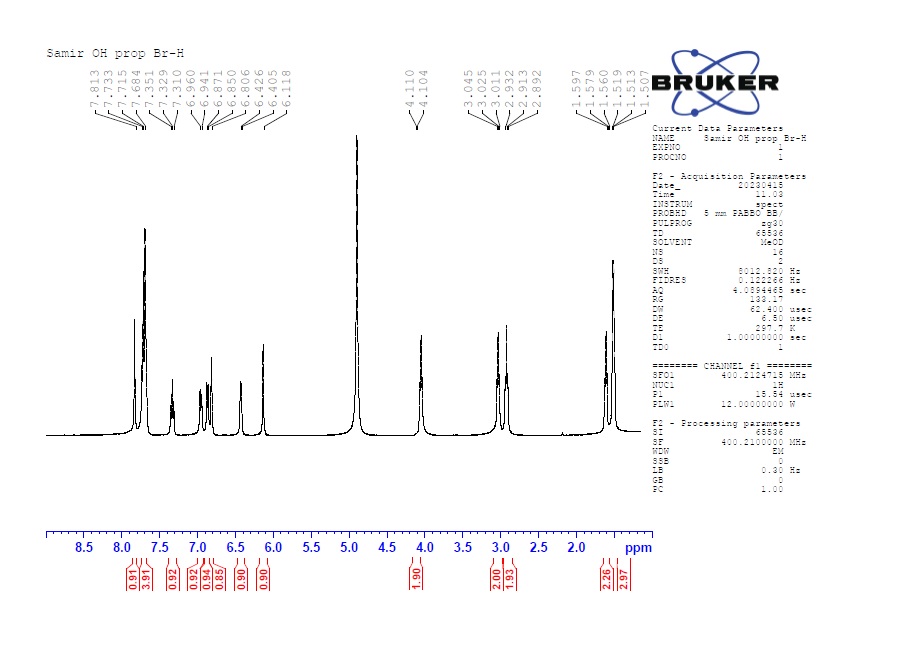

**14b**

**14b**


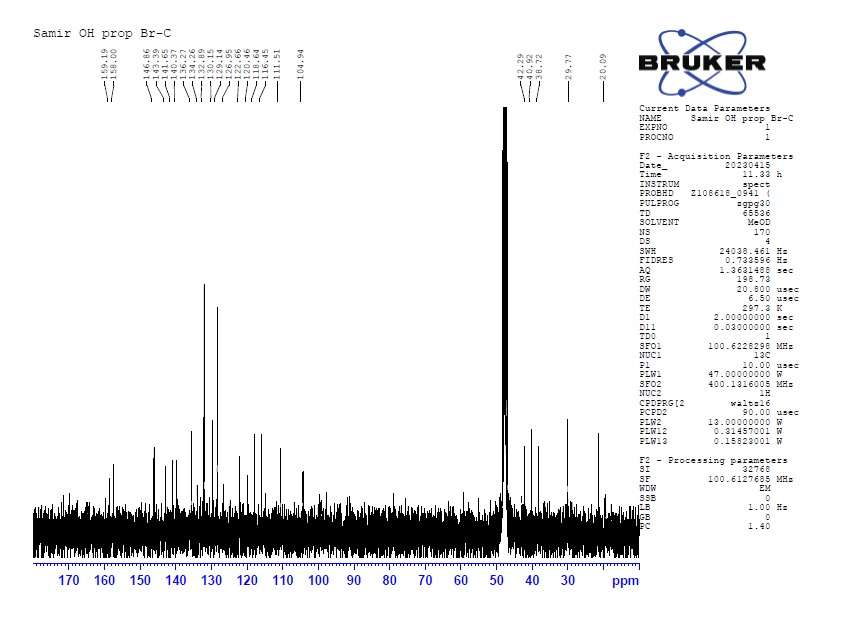

**14b**


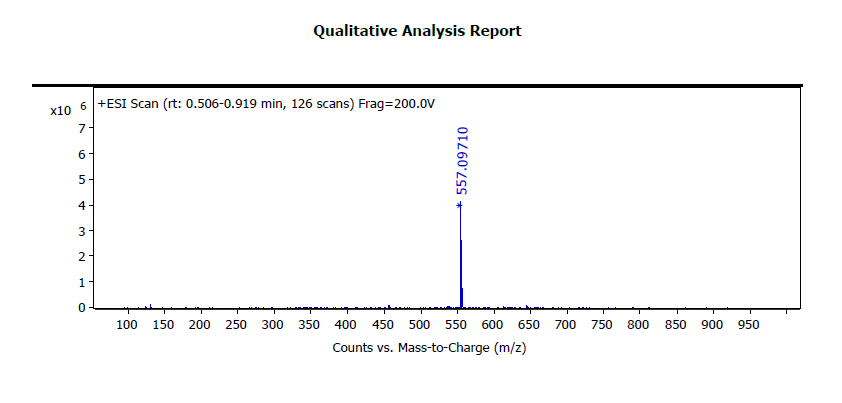

**14c**


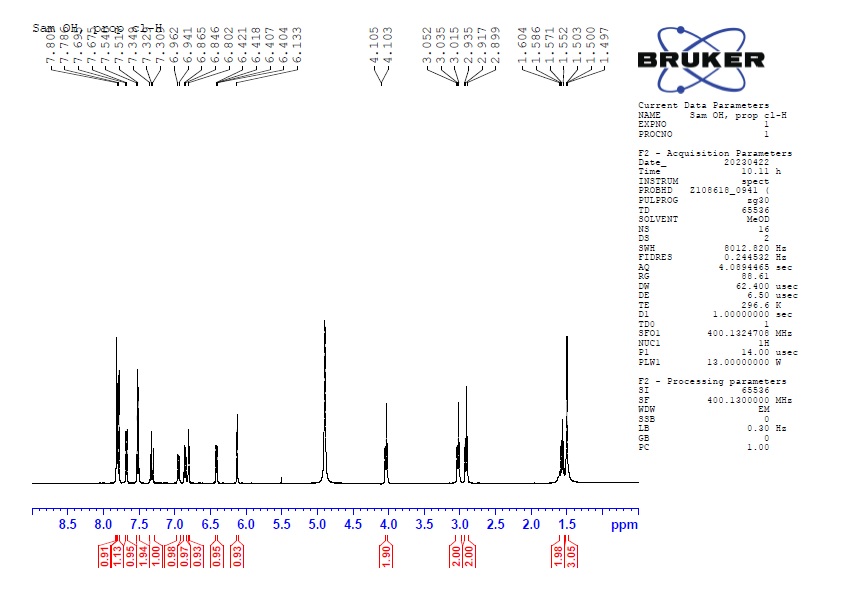

**14c**

**14c**


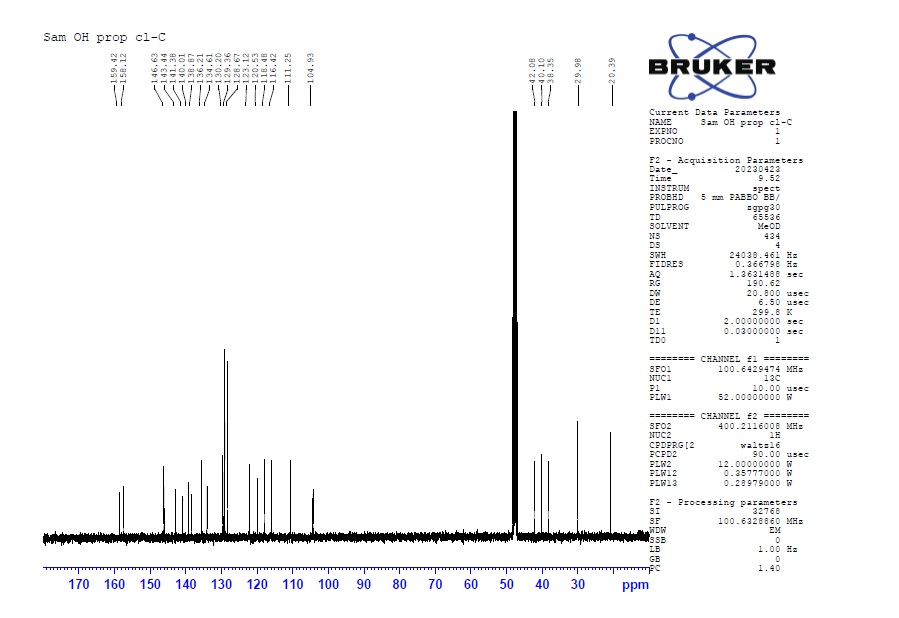

**14c**


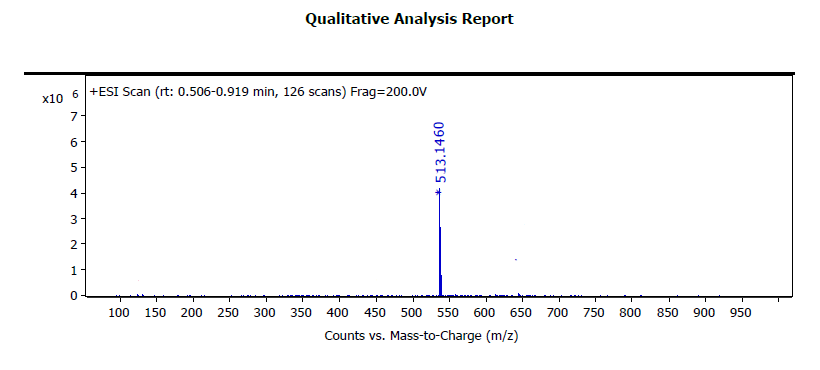

**14d**


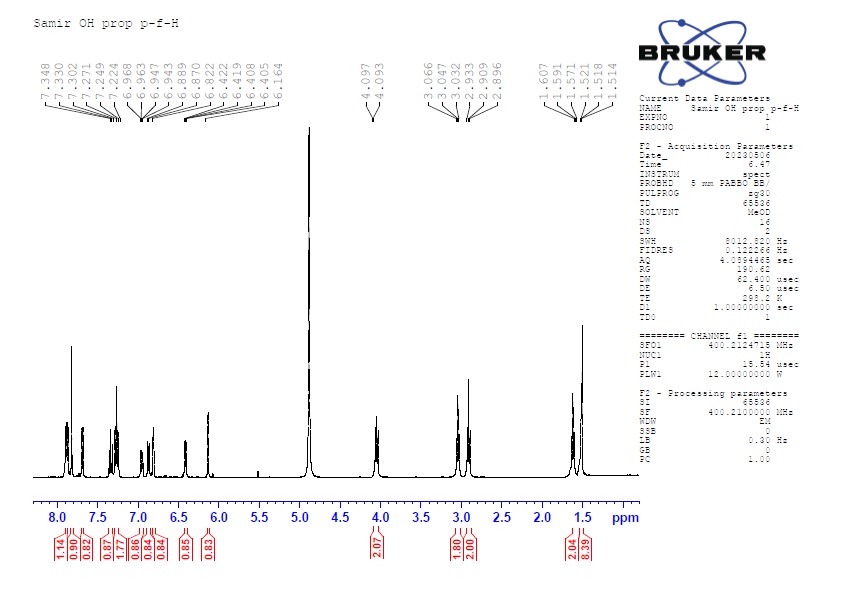

**14d**


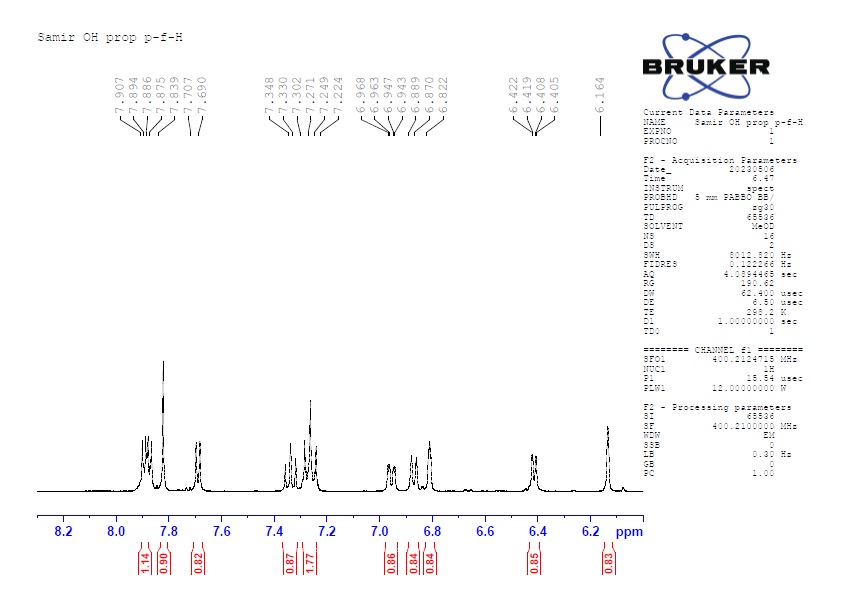

**14d**


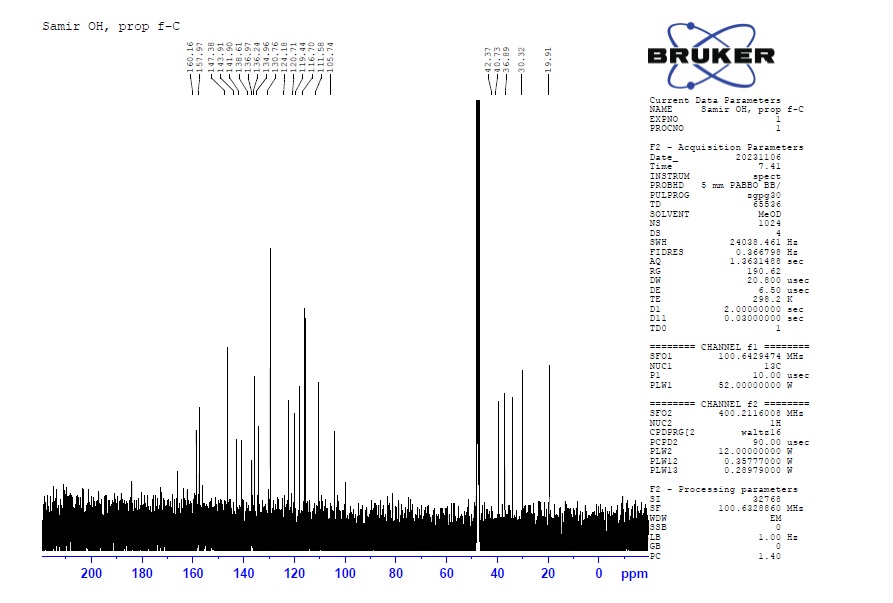

**14e**


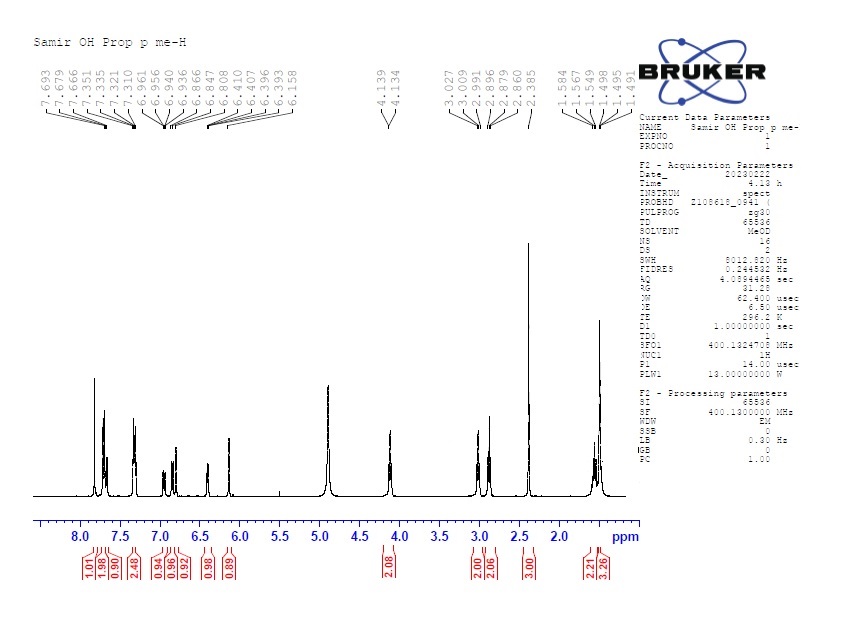

**14e**

**14e**


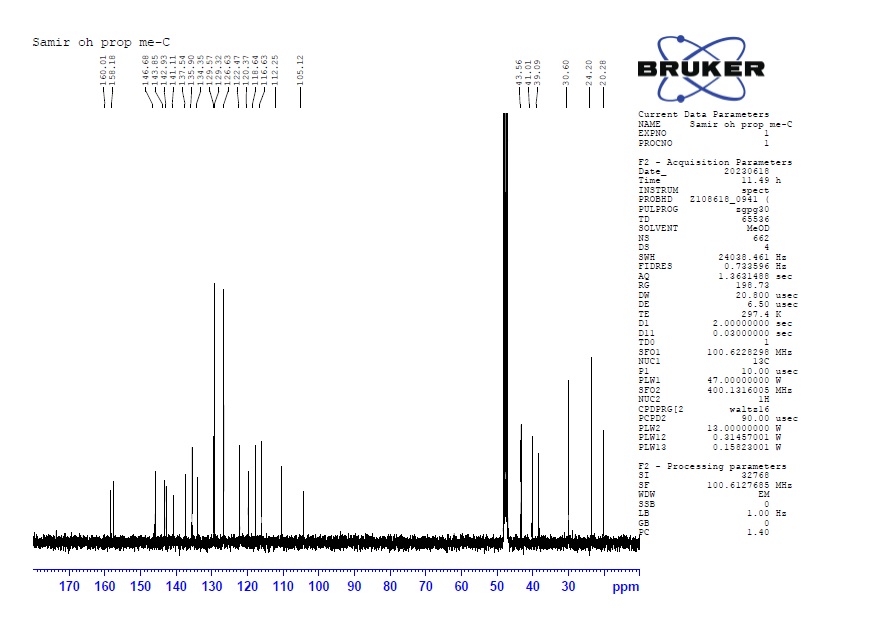

**14f**


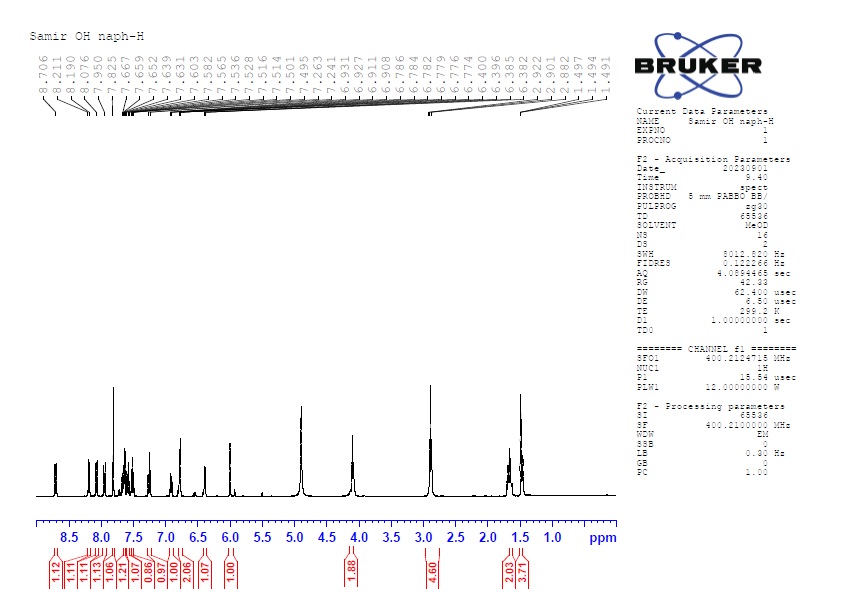

**14f**


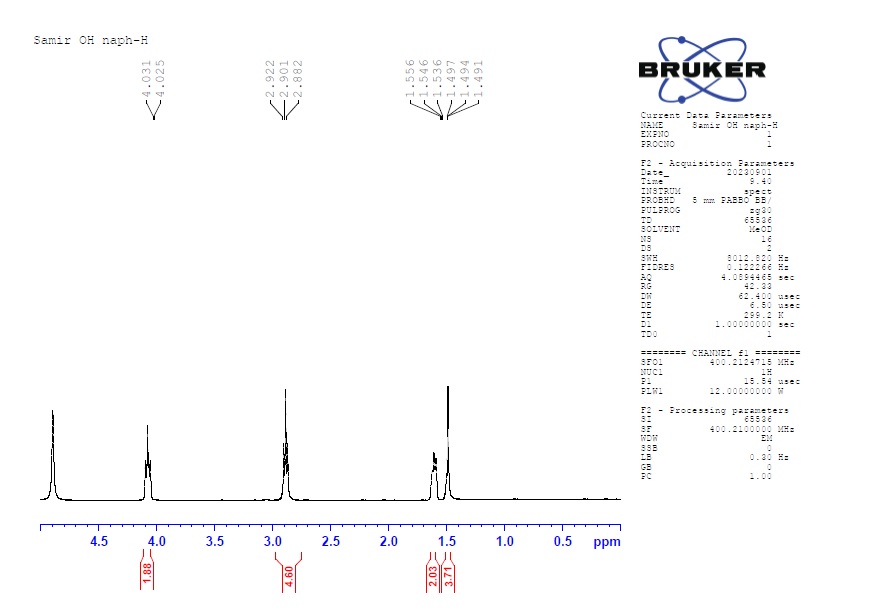

**14f**

**14f**


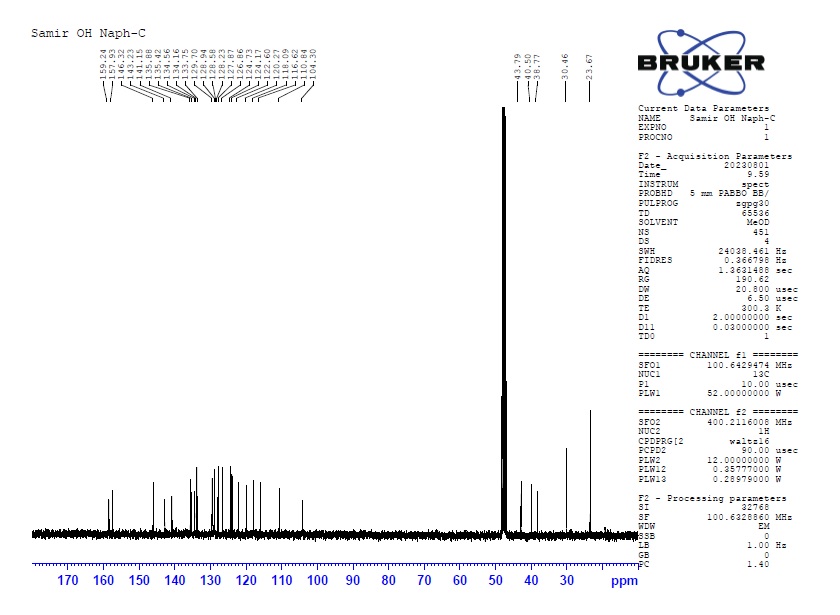

Supplement: Supplementary file 1 — Supplementary file1 (DOCX 5063 KB) [file 11030_2025_11121_MOESM1_ESM.docx]
